# From Genome Sequencing to CRISPR-Based Genome Editing for Climate-Resilient Forest Trees

**DOI:** 10.3390/ijms23020966

**Published:** 2022-01-16

**Authors:** Hieu Xuan Cao, Giang Thi Ha Vu, Oliver Gailing

**Affiliations:** 1 Forest Genetics and Forest Tree Breeding, Georg-August University of Göttingen, Büsgenweg 2, 37077 Gottingen, Germany; xuanhieu.cao@uni-goettingen.de; 2 Center for Integrated Breeding Research (CiBreed), Georg-August University of Göttingen, 37073 Gottingen, Germany

**Keywords:** CRISPR-Cas, forestry, genome engineering, climate change, tree genomes

## Abstract

Due to the economic and ecological importance of forest trees, modern breeding and genetic manipulation of forest trees have become increasingly prevalent. The CRISPR-based technology provides a versatile, powerful, and widely accepted tool for analyzing gene function and precise genetic modification in virtually any species but remains largely unexplored in forest species. Rapidly accumulating genetic and genomic resources for forest trees enabled the identification of numerous genes and biological processes that are associated with important traits such as wood quality, drought, or pest resistance, facilitating the selection of suitable gene editing targets. Here, we introduce and discuss the latest progress, opportunities, and challenges of genome sequencing and editing for improving forest sustainability.

## 1. Introduction

Forests are of critical importance ecologically and economically. They cover more than one-quarter of the Earth’s land surface area, harbor the majority of the terrestrial biodiversity [1,2], exert strong control on biosphere carbon sinks [3], have a pivotal role in climate regulation [4], and are widely acknowledged as being principle ecosystem service providers (for review, see [5]). Global climate change, with longer droughts and higher temperatures, produces strong impacts on forest trees [6], altering future species distributions [7] and subsequently the structure and functioning of forest ecosystems [8,9]. Increasing adaptability of forest trees to abiotic stress factors and resistance to pests, diseases, and herbicides as well as improving the timber productivity and wood quality have become essential to advance not just productivity of economically important species, but also climate resilience, forest health, and conservation.

CRISPR (clustered regularly interspaced short palindromic repeats) technology, the recent system of choice for targeted mutagenesis, was discovered by the identification of a family of prokaryotic endonucleases that use programmable RNAs for site-specific DNA cleavage in virtually any species [10,11,12]. The high accuracy, simplicity, and efficiency of the CRISPR systems for targeted DNA mutations are behind the current revolution in genomic editing in plant breeding, including woody trees (for review, see [13,14,15]). However, several aspects affect their various applications in plant systems, including the activity of Cas nucleases, target site selection, guide RNA design, delivery methods, off-target effects, and the incidence of DNA repair outcomes. This review will highlight current advances of the technology as well as possible strategies for handling any typical problems in forest tree systems.

**Figure 1:**
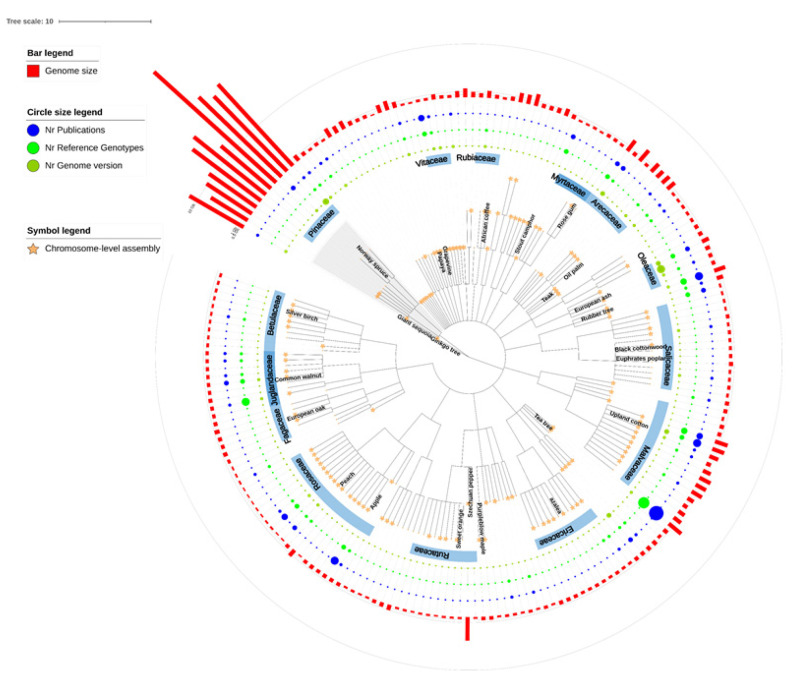
Published reference genomes of trees and woody plant species. Phylogenetic relationships of 201 species were extracted from NCBI Taxonomy and displayed by using iTOL tool [19]. The gymnosperm clade is labeled in the grey background and the important tree families are highlighted. Star symbols indicate 125 species with chromosome-level reference genomes. Numbers of genome versions, genotype-specific reference genomes, and genome publications are visualized as sizing circles. The estimated genome sizes of species are shown as red bars in the outermost circle. Detailed data is available in Table S1.

Forest trees differ from herbaceous, annual model plants or crops by their perennial growth habit and long life span. They can, and usually do, live for many decades with long generation times [16]. In addition, they are unique because of their ability to form secondary xylem, or woody stems, supporting their growth from several up to a hundred meters in height. The tree or woody phenotype evolved, for the first time over 300 million years ago (mya), in many plant families with nowadays about 60,000 tree species that are distributed around the world [17,18]. Woody perennial plants can be found in two main groups of seed-producing plants: Gymnospermae (sometimes referred to as coniferous trees or softwoods) and Angiospermae (sometimes referred to as broad-leaved trees, non-coniferous trees, or hardwoods). Furthermore, several forest tree species have exceptionally large and complex genomes relative to other plant species. Thanks to major advances in sequencing technology (i.e., massively parallel DNA sequencing, long-read sequencing, sequence extension technologies), the reference genomes of almost 700 plant species have been published (http://www.plabipd.de accessed on 19 December 2021), including 200 trees or woody plants (Figure 1). Given that genomics and CRISPR technology have been evolving rapidly in recent years, in this review we highlight the latest genomic resources of forest tree species and elucidate the impact of whole-genome sequencing, omics studies, and genome editing on future basic and applied research in this diverse group of plants.

## 2. High-Quality Reference Genomes and Extensive Genome-Wide Genetic Resources Available for Forest Trees

Four years after the first plant genome was sequenced (*Arabidopsis thaliana* with a genome size of 150 Mbp), the black cottonwood (*Populus trichocarpa*, or poplar) was the first tree to have its genome sequenced, because of its widespread use as the model tree species and a potential source of renewable energy [20]. The black cotton genome (485 Mbp, which is relatively small to other widely planted woody species) was sequenced by the Sanger sequencing approach and assembled using a strategy that combined WGS sequencing with BAC-end sequencing and high-density genetic mapping. The reference genome of this “model tree” was critical to complement and integrate the pre-existing genetic resources (i.e., genetic maps, transcriptomes) of other closely-related tree species. In addition, the phylogenetic proximity of *Populus* spp. to *A. thaliana* in the Eurosid clade (diverged ca. 100–120 mya) also offered an opportunity for comparative analysis of two eudicot models with strongly contrasting life histories and adaptations, revealing that many of the molecular mechanisms and genes are largely shared [21].

Since 2010, with advances in massive parallel DNA sequencing, the sequencing cost has exponentially decreased, accelerating progress in whole-genome sequencing. Consequently, genome sequences of about 200 forest tree species (Figure 1, Supplemental Table S1) from almost 30 taxonomic orders, covering the most important forest tree families, such as Pinaceae (pines, spruces, and firs), Salicaceae (poplars and willows), Myrtaceae (eucalyptus), and Fagaceae (oaks, chestnuts, and beeches) have been reported. Most of the recently published tree genomes were sequenced by using one or more next-generation sequencing platforms. Recent progress in long-read sequencing, together with high-throughput long-range technologies (e.g., optical mapping, chromosomal conformation sequencing) has contributed to clustering the assemblies into chromosome-sized pseudomolecules, enabling a rapid increase in the number of high-quality and chromosome-level published genomes in forestry.

Over the last few decades, genomics has generated an exceptional transformation in the way genetics is studied in biology, and particularly in forestry. High-quality reference genome sequences and extensive genomic resources are nowadays the keys to the discovery of genes and biological processes that are associated with adaptive traits of interest, such as bud burst, drought resistance, and complex traits of economic importance such as fruit and wood quality [22,23]. Since typical forest tree populations consist of long-lived, outcrossing and genetically diverse individuals, it is crucial to collect and store the metadata on the phenotypic and environmental metrics that are associated with the sequenced trees and their georeferenced populations. Furthermore, to address questions that are related to tree breeding and forest health, several tree database cyberinfrastructures have been established not only for supporting comparative genomics, population genetics, expression profiling, and genome annotation; but also for keeping pace with the magnitude of genomic and phenomic sampling of larger populations. There are several tree or forest tree resources (e.g., TreeGenes [24], Hardwood Genomics Project [25], PlantGenIE-based platforms [26]) that focus on a combination of model and non-model systems and integrate with established comparative resources (e.g., Phytozome [27], PLAZA [28], and Planteome [29]) to deliver value-added information (for review, see [30]). For instance, TreeGenes currently curates 38 reference tree genomes together with genomic, transcriptomic, and phenotypic data for more than 2000 species representing 227 genera and 16 orders. The future of forest tree databases may involve the integration and annotation of millions of genotypes across thousands of individuals or hundreds of pan-genomes with the associated metadata.

## 3. Approaches for Sequencing of the Large and Heterozygous Tree Genomes

Despite immense advancements in high-throughput DNA sequencing technologies, the vast majority of tree genomes, and in particular, forest trees, remain elusive. Among only 200 of the few tens of thousands of known tree species that have a reference genome sequence, many were firstly chosen due to their relatively small genome size, besides their importance to humans and their scientific significance. Several forest tree species have exceptionally large and complex genomes, especially gymnosperm (conifer) species with ~20 Gbp or larger genomes. It is worthy to note that mating designs (i.e., selfing) commonly used in crops and model systems are not feasible or difficult in forest trees because of high genetic loads and long generation times. For instance, many conifer species require several decades to reach sexual maturity. To avoid the high heterozygosity in somatic cells of long-lived trees, one approach that was used for conifer genomes (e.g., Norway spruce (*Picea abies* L., 20 Gbp) [31] and loblolly pine (*Pinus taeda* L., 22 Gbp) [32]) was taking advantage of conifer seeds with a large haploid (1N) megagametophyte for providing DNA material. Alternatively, DNA from *Taxus chinensis* endosperm calli [33] containing haploid chromosomes (~10 Gbp in size) was used for whole-genome sequencing through Illumina (Illumina Inc., San Diego, US), Pacbio single-molecule real-time sequencing (Pacific Biosciences of California, Inc., Menlo Park, US), and Hi-C libraries. For the case of the 640-Mbp genome sequencing of flooded gum (*Eucalyptus grandis*, [34]), a genotype that was derived from one generation of selfing was used to mitigate the challenge of assembling a highly heterozygous eucalypt genome.

A high proportion (50% or more) of repetitive sequences in the large plant genomes [35] can cause serious difficulties for the de novo genome assembly from Illumina sequencing reads. The short reads (150 bp and 300 bp read length for HiSeq/NextSeq and MiSeq platforms, respectively) cannot fully span the repeat regions, resulting in fragmented sequence contigs and incomplete genome assembly. The unassembled (and therefore missing) sequences in a draft genome may consist of complete genes, partial regulatory elements, centromeres, and telomeres which are of biological significance for understanding genome structure and function. Alternatively, long-read sequencing platforms (i.e., PacBio SMRT sequencing; Oxford Nanopore technology (Oxford, UK)) generate reads with a read length of 15 kb or up to hundreds of kilobases, enabling relevant assemblers to resolve repeat regions including centromere and telomere tracts. However, due to relatively high error rates (in comparison with Sanger or Illumina sequencing) of the long-read sequencing technologies, a high sequencing depth (corresponding to higher sequencing cost for ca. 50×–100× genome coverage) or the combination with short-read sequencing (~200× genome coverage) for error correction is required. The latter approach could be more suitable for sequencing very large genomes or improving draft genomes. Nevertheless, PacBio and Oxford Nanopore companies have actively upgraded their chemistry, software and devices for improving accuracy rates (>99%) and yields (up to megabase read length).

Although the impact of new sequencing technologies is certainly evident, it has not been possible to assemble a gap-free genome from telomere to telomere only by short-read sequencing. In more recent genome sequencing projects with high-quality published genomes [36,37,38,39,40,41], the scaffolding of long-read assemblies was complemented with high-throughput long-range data, such as optical maps (BioNano Genomics, San Diego, CA, USA [42,43]), chromosomal conformation sequencing (Hi-C with in vivo fixation of chromosomes [44,45]), or linked-reads sequencing (10× Genomics, 10× Genomics, Inc., Pleasanton, US [46]). Long-range mapping data and long-read sequencing allow haplotype/subgenome-phasing of several tree genomes [47,48,49], solving, to a certain extent, challenges in the complete assembly of highly heterozygous and complex genomes. Importantly, even if long-range technologies make it possible to organize contigs into chromosome-sized pseudomolecules, they are often not able to fill the sequencing gaps between these contigs. The remaining imperfections of assemblies, which are largely due to the complexity and heterozygosity of the sequenced genomes, require higher sequence coverage, longer sequencing reads, and/or additional scaffolding and haplotype/subgenome-phasing information. High-quality genome assemblies (i.e., high Phred base accuracy Q > 50) are suitable for use in phylogenomics and population-scale SNP surveys, even though the assembly continuity is relatively low (i.e., 10 kilobases of contig N50 and several megabases of scaffold N50). Instead, to perform a chromosomal evolution study of a genome, higher continuity of the assembly is necessary and more than 95% of the assembly length needs to be assigned to its chromosomes. Recently, a telomere–telomere gapless chromosomal assembly of homozygous banana (*Musa acuminate*, with a medium-size ~500 Mbp genome) has been reported using 177× genome coverage of Oxford Nanopore long-read sequencing [50]. The further improvements of the long-read technology (e.g., base-call accuracy), coupled with the evolution of the bioinformatics tools (i.e., more accurate and complete haplotype phasing, better resolution of long repetitive tracts), high-molecular-weight DNA extraction protocols, and new double-haploid technology (for obtaining homozygous DNA material) will pave the way for high-quality chromosome-scale genome assemblies for tree species.

Altogether, the sequencing and high-throughput mapping costs for a high-quality and/or chromosome-level reference genome of these giant-genome plants are still high, requiring collective funding and resources from international genome sequencing consortia rather than from a single project or a laboratory. Moreover, due to the high degree of genomic plasticity in tree species, single reference genomes do not represent the diversity within a species. In addition to core genes that are found in all individuals, variable genes (i.e., that are absent in individuals of certain populations) are commonly enriched and associated with resistance to stress and pathogens [51,52]. A surge in advanced genome sequencing technologies is expected to facilitate the de novo assembly of pan-genomes from chromosome-level genome sequences of multiple genotypes or individuals, exploring structural variants as well as the origins of gene presence and absence variation in tree breeding and local adaptation studies.

## 4. Distinctive Features of the Tree Genomes Sequenced

In plant science, *Arabidopsis thaliana* has been adopted as the prime model system with an impressive number of tools, data, and techniques that are now available to understand the gene functions in this plant. However, in many physiological and genetic respects, *Arabidopsis* is a highly specialized plant and a genetic extreme in terms of its small genome size. The striking differences in appearance and physiology of different plant species show that a single model cannot be used to answer all biological questions. For instance, the highly accelerated life cycle of *Arabidopsis* makes many traits that are essential in many plants unimportant in *Arabidopsis*; two obvious examples are wood formation and seasonality of growth. As an opposite extreme model, forest trees have long life spans and generation times, and woody perennial growth habits. Therefore, tree genomes provide opportunities to study important plant processes that are absent or poorly developed in Arabidopsis or other herbaceous, annual model plants.

### 4.1. Slower Evolutionary Clock in Trees

Sequencing of the first woody perennial tree genome—black cottonwood—resulted in several discoveries that are relevant to understanding the genome evolution of other woody perennial species. For instance, the sequence divergence that was observed between paralogous genes that were derived from the most recent duplication (salicoid duplication) of the *Populus* genome was estimated to have occurred at 8–13 mya, based on the molecular clock with synonymous rates that are commonly used for the analysis of annual plants [20]. However, the fossil record shows that this duplication event was as far more ancient as 60–65 mya. The most plausible explanation for this discrepancy is a slower evolutionary clock in *Populus*. Similar evidence for a slower evolutionary clock (i.e., low synonymous substitution rate) has also been reported for other tree species, such as those within *Eucalyptus* [34] and *Pinus-Picea* [31,53]. An evolutionary study for five major angiosperm branches (i.e., Apiales, Commellinidae, Moraceae—Urticaceae, Primilales, and Dipsacales) demonstrates that evolutionary clocks are consistently slow in trees and shrubs, which generally have relatively long generation times, as compared with related herbaceous plants with shorter generation times [54]. A slower clock in woody species could be due to the long-lived perennial status, leading to recurrent contribution of “ancient” gametes from old individuals. Besides, the generation time, genome size, plant height, and DNA repair system could be additional life-history factors attributing to the lineage-specific variation of evolutionary clocks as shown in several phylogenetic studies on a wide range of taxonomic families [55,56]. It is important to note that the main driving mechanisms of variation in the molecular evolutionary rate are not entirely understood, although our understanding of evolutionary clocks has recently been aided by the growing availability of genomic sequence data.

### 4.2. Plasticity of Gene Content in Tree Genomes and the Contributions of Whole Genome Duplication, Tandem Duplication, and Repetitive Amplification

Given both the smallest (60–80 Mbp, *Genlisea* spp. [57]) and the largest (149 Gbp in *Paris japonica* [35]) plant genomes that were ever found belong to herbaceous species, the genome size variation in tree species is smaller. The largest tree genome that was sequenced belongs to the sugar pine (*Pinus lambertiana* with 31 Gbp [58]), while the smallest tree genomes that were sequenced are 221 Mb in Indian sandalwood (*Santalum album*, an important tropical evergreen tree [59]) and 265 Mbp in peach (*Prunus persica*, a highly genetically characterized deciduous tree [60]).

It is believed that genome size variation is independent of gene content and organism complexity, known as the “C-value paradox” or “C-value enigma” [61,62,63]. The high-confidence gene numbers in sequenced trees, predicted around 30,000 to 40,000 regardless of the genome size, confirm this observation. The annotation of *Populus trichocarpa* genome assembly (410 Mbp [20]) uncovered ca. 45,500 putative protein-coding genes, significantly more than in *A. thaliana* (150 Mbp, 27,206 protein-coding genes, and 33,323 nuclear genes in total [64]). This large gene number in poplar has possibly resulted from a recent whole-genome duplication (WGD) 60–65 mya after the divergence from the *Arabidopsis* lineage (ca. 100–120 mya [65]). WGD has been considered a significant driver for the diversification and key innovations in plant species [66]. A large number of coding sequences with new or additional functions (in the processes named as neofunctionalization and subfunctionalization of retained paralog genes) is owed to the obvious need for survival and adaptation underlying strongly contrasting life histories between the two model plants. The genome of the most cultivated hardwood species worldwide, *Eucalyptus grandis,* was sequenced with a total length of ca. 640 Mbp and 36,376 predicted protein-coding loci [34]. In the evolutionary history of the eucalypt genome, there was a lineage-specific WGD ca. 110 Mya. However, in comparative analysis with the basal rosid lineage, represented by the *Vitis vinifera* genome [67], most (>95%) of the paralogues in *Eucalyptus* have been lost after the WGD. Strikingly, 34% of eucalypt genes appeared in tandem repeats while the corresponding 18% were found both in *A. thaliana* and *P. trichocarpa*. Tandemly-duplicated genes are often involved in stress responses, suggesting that they may be related to the adaptive evolution of *Eucalyptus* in diverse environments. Interestingly, the pattern of tandem duplications appears to be dynamic even within the genus *Eucalyptus*, pointing to high genome plasticity. Not surprisingly, the *E. grandis* genome contains the largest number (*n* = 113 compared to *n* = 34 in *A. thaliana*, 59 in *P. trichocarpa*, or 83 in *V. vinifera*) of terpene synthase genes ever reported. An extremely diverse array of secondary metabolites was observed in *Eucalyptus* spp. [68,69], leading to high disease and insect resistance of eucalyptus trees.

The smallest tree genomes that were sequenced were of Indian sandalwood (*Santalum album* [59]) and peach (*Prunus persica*, an important fruit tree with a ca. 4000-year domestication and intensive breeding [60]) containing 38,119 and 27,852 putative protein-coding genes, respectively. Although comparative analysis showed the absence of any recent WGD in the peach genome, the genomic data support the massive polyol biosynthesis and accumulation as being linked, in part, to gene number expansion in particular gene families. Furthermore, based on expanded gene families that were derived from whole-genome resequencing of different *P. persica* accessions and wild peach relatives (i.e., *P. mira*, *P. kansuensis*, *P. davidiana,* and *P. ferganensis*) [70], high nitrogen recovery was proposed as an important factor for high-altitude adaptation of *P. mira* through increasing its resistance to low temperature.

In the above 3-Gbp genomes of the tea plant (*Camellia sinensis,* evergreen shrubs, or trees are commercially cultivated across the world), ~40,000 protein-coding genes were predicted according to several current assemblies. The chromosome-level genomes of four elite cultivars ([71,72,73], especially a haplotype-resolved assembly [48]) and an ancient tree [74] were recently released, representing one of the largest chromosome-level high-quality genomes of angiosperm perennial and woody plants. More than 70% of the tea genome comprises repetitive sequences, among which, LTR transposable elements represent a large proportion (ca. 53% of the genome). Evidence in tea genomes showed that LTR-RTs play critical roles not only in genome size expansion (i.e., by an incessant burst event of a handful of LTR-RT families during the last 1 mya that accounted for ~30% of the genome [73]) but also in the transcriptional diversification of tea plant genes through preferential insertion in promoter regions and introns [71]. Similar to the case of the *Eucalyptus* genome, genes encoding terpene biosynthesis, associated with tea’s pleasant aroma and biotic stress resistance, were significantly enriched proteins (*n* = 72, compared to *n* = 34, 53, 36, and 45 in kiwifruit [75], coffee [76], cacao [77], and the most recent common ancestor, respectively) through recent tandem duplications and present as gene clusters in the tea plant genome. Interestingly, caffeine (1,3,7-trimethylxanthine), one of the most well-known alkaloids in plants, is synthesized by several eudicot woody plants such as tea (*Camellia sinensis* from the asterids family Theaceae), coffee (*Coffea arabica* from the asterids family Rubiaceae), and cacao (*Theobroma cacao* from the rosids family Malvaceae). The tea genomes suggest that tea plants have experienced two rounds of WGD events, one with the core-eudicot whole-genome triplication (WGT-γ) and the most recent event shared by the Polemonioids-Primuloids-Core Ericales sections [78]. These WGDs were followed by extensive genomic rearrangements with a rapid gene and genome evolution in tea plants. Indeed, approximately half of the duplicated genes that are located in collinear genomic blocks with closely-related plants lost their duplicated copy after the recent WGD. Furthermore, about 25% of the retained duplicates, mainly including genes that are related to the secondary metabolic process, diverged rapidly through mechanisms such as expression divergence, neofunctionalization, and subfunctionalization. Importantly, population genomic analysis using genomic data of 190 *Camellia* accessions revealed independent evolutionary histories and parallel domestication in two widely cultivated varieties, var. *sinesis* (CSS, Chinese type) and var. *assamica*. (CSA; Assam type). Strong signatures of artificial selection were associated with biosynthetic and metabolic pathways that contribute to various aromatic chemicals, cold tolerance, and different plant heights [48]. For instance, two cytochrome P450 genes that are involved in brassinosteroid biosynthesis, photomorphogenesis, and dwarfism were under artificial selection in cultivated varieties that are likely associated with the reduction of plant height (wild tea plants in the forest can reach more than 4 m in height), with CSA being small trees or semi-shrubs and CSS being shrubs.

Gymnosperm plants are unique in that their genome sizes are much larger than those of most other plants [79]. Norway spruce (*Picea abies*, as one of the most economically important forest tree species), with a genome of ca. 19.6 Gbp, became the first gymnosperm to be sequenced [31], followed by white spruce (*Picea glauca*, 20.8 Gbp [80,81]), loblolly pine (*Pinus taeda*, ~22 Gbp [32,82]), sugar pine (*Pinus lambertiana*, 34.1 Gbp [58]), Douglas-fir (*Pseudotsuga menziesii*, ~16 Gbp [83]), Silver fir (*Abies alba*, ~18.2 Gbp), and Siberian larch (*Larix sibirica*, 12.3 Gbp [84]). Due to the large genome size and high repetitive sequence content (mostly >70% genome) in these gymnosperm trees, it is a challenge to obtain high-quality reference genomes and accurate annotation of protein-coding genes. However, the first high-quality gymnosperm reference genome has just recently been reported for *Ginkgo biloba* [41] with a genome size of ~10 Gbp and 27,832 protein-coding genes. The data suggest that gymnosperms do not have a significantly larger number of protein-coding genes (classified as high-confident genes supported by transcript and/or homology evidence) than angiosperms, although pseudogenes are abundant and introns are greatly expanded in length and inserted by repeat elements. Notably, the genome of the extant *G. biloba* had undergone the common seed plant WGD (known as zeta WGD ~310 mya [85]), but no additional round of lineage-specific WGD recurred during the evolutionary process.

### 4.3. Growth Forms between Woody Tree, Shrub, and Liana

Recent years have seen a surge in plant genome sequencing projects, enabling the comparison of genomes from multiple related species and taxonomic lineages. Among five distinct lineages of today’s seed plants, including the species-rich angiosperms and four gymnosperm lineages, gnetophytes represent an ancient, enigmatic, non-tree gymnosperm lineage differing from other extant gymnosperms in growth forms, such as the shrub and liana habit and specialized leaf morphologies of *Gnetum*. The genome of *Gnetum montanum* (with ~4.2 Gbp in size and encoding 27,491 protein-coding genes, [86]) showed a large expansion of the CslB/H subfamily of cellulose synthases (*n* = 20), many of which were differentially expressed in leaves, stems, and roots. In contrast, only one to six genes of this cellulose synthase subfamily were found in other species that were analyzed including *Picea abies* (*n* = 5) and *Pinus taeda* (*n* = 1).

The higher the phylogenetic relatedness between the compared species/taxa with contrasting features, the more the different patterns of gene loss, retention, and amplification may be associated with their distinctive forms and life history strategies. Willows (*Salix* with >300 species) and poplars (*Populus* with ~29 species), diverged from each other around the early Eocene ~60 mya, are known worldwide as woody species with diverse uses. Poplars generally have the form of large trees, while willows exhibit different growth forms, including large trees, subtrees, and small shrubs. These two genera share numerous traits, including the same chromosome number of 2n = 38 and the common ‘Salicoid’ genome duplication with a high macrosynteny [87]. Chromosome-scale assembly of *Salix suchowensis*, an early-flowering shrub willow, was generated with a total length of 356 Mbp and 36,937 protein-coding genes [88]. A stronger purifying selection was observed for each chromosome in *S. suchowensis* than in *P. trichocarpa*, leading to a faster loss of duplicated genes in willow than in poplar. Comparative analysis among gene families that are involved in cellulose and hemicellulose biosynthesis between *S. suchowensis*, *P. trichocarpa*, and *Arabidopsis thaliana* suggested that glycoside hydrolase (GH) and lignin biosynthesis genes were enriched in *S. suchowensis* (*n* = 275 and 75, respectively) and *P. trichocarpa* (*n* = 272 and 90) as compared to *A. thaliana* (*n* = 242 and 34). Particularly, caffeic acid O-methyltransferase (COMT) was proposed as a potential target enzyme for modifying the composition of lignin in plants that have 9 and 13 copies in *S. suchowensis*, *P. trichocarpa*, respectively, but a single-copy in *A. thaliana*.

## 5. CRISPR-Mediated Genome Editing Provides a Powerful Tool for Forest Tree Improvement

Because of many advantages in simplicity, efficiency, precision editing, a wide range of accessible targets, cost-effectiveness, and robustness, CRISPR-based genome editing has enormous impact and wide-ranging applications in all principal branches of eukaryotic organisms (for reviews on potential applications, see [13,89]). There are two components of the engineered CRISPR system: the RNA-guided endonuclease (RGEN) and the single-guide RNA (sgRNA), both can be included and are deliverable as a single plasmid. Among the various bacterial RGEN, the Type II Cas9 from *Streptococcus pyogenes* (SpCas9) has been widely adopted for genome editing (GE) in many organisms, including trees. The sgRNA is a short synthetic RNA that is composed of a 17–20 nucleotide sequence that is homologous to the target genomic regions of interest (called a protospacer). A prerequisite for the programmable cleavage of the target DNA by the SpCas9 endonuclease is the presence of a sequence 5′-NGG-3′ or 5′-NAG-3′ as the conserved protospacer-adjacent motif (PAM). The SpCas9, when forming a ribonucleoprotein complex (RNP) with sgRNA, produces double-strand breaks (DSB) at the target DNA region, permitting target-specific mutagenesis. The sequence context (i.e., the presence and arrangement of repeats) around the DSB and the spatial and temporal availability of the cellular DNA repair machinery (in other words, the cell cycle and the genetic background of the target organism) determine the repair pathway that is used, and thus, the outcome of DSB repair [90]. The non-homologous end joining (NHEJ) repair pathway is the most active repair mechanism and it frequently causes a broad spectrum of small nucleotide deletions or insertions of short stretches of nucleotides. When the DSB occurs within a coding sequence, the resulting InDels often cause a frameshift mutation or a premature stop codon, leading to loss-of-function (i.e., knockout, KO) mutations of the targeted protein-coding gene. By contrast, and at a much lower efficiency, repair by homology-directed repair (HDR) can generate more precise modifications including insertion of a sequence of interest (a transgene integration or knock-in replacement) by typically introducing an exogenous DNA repair template.

There are several points that one needs to consider for designing CRISPR experiments: (1) the applicable and efficient delivery method for CRISPR reagents (via DNA plasmid, mRNA, or RGEN-gRNA (RNP) protein); (2) suitable CRISPR reagents, including RGEN, promoters controlling expression levels of RGEN and sgRNA in the DNA plasmid delivery format), and the optimal cloning strategy; (3) designing one or more sgRNA for targeting genes or genomic regions of interest; (4) the corresponding and appropriate screening or selection strategy for the desirable edited plants (for a review on technical and practical details, see [91,92]). With the AddGene Repository [93] that deposits and shares more than 9000 CRISPR-related plasmids (out of a total ~100,000 plasmids) including plant-specific plasmids and toolkits, laboratories from around the world have been able to start designing and carrying out CRISPR genome engineering experiments. In addition, there are dozens of bioinformatics tools that are available to optimize gRNA design, detect off-target regions, and in silico design the assembly of the constructs to be used for plant transformation. The most commonly used tools are CRISPR-P 2.0 [94], Cas-Designer [95], Cas-OFFinder [96], ZiFiT Targeter v 4.2 [97], CasOT [98], E-CRISP [99], GoldenBraid 3.0 [100], and CRISPOR [101]. The sequencing results of the edited plants can be analyzed by manual screening or by using online tools such as TIDE [102], CRISPResso2 [103], or ICE [104]. Although endogenous sequence patterns have been shown to predispose the repair modes of CRISPR/Cas9-induced DNA DSB in *A. thaliana* [90], so far there is only one predictor tool, FORECasT [105], using human data and limited to 30-bp mutations for predicting the mutations generated by repair of CRISPR/Cas-induced DSBs. The up-to-date list of gRNA design tools as well as educational guidelines for CRISPR experiments can be found on the AddGene website (www.addgene.org/crispr/reference/ accessed on 19 December 2021). For non-model species for which reference genomes have not been publicly available, custom bioinformatic approaches need to be developed, possibly including (1) detection of sgRNA sequence candidates with required PAM in the target genes, (i.e., following the suggestions from [106,107,108,109,110,111] for optimizing sgRNA structures); (2) screening for the specific and homologous (allelic variant-free) sgRNA sequences by blasting the 18–20 nucleotide sequence upstream the PAM to the available reference genomes or transcriptomes of closely related model plants; and (3) the validation of the homologous sgRNA in the genome of interest by specific amplification and sequencing from the genomic DNA of the study species. Given the entire process of stable transformation will normally be labor-intensive and time-consuming in trees, it is advisable to further validate the functionality of sgRNAs using an applicable transient expression system, such as an in vivo CRISPR/Cas9-mediated protoplast or hairy root genome editing.

**Figure 2:**
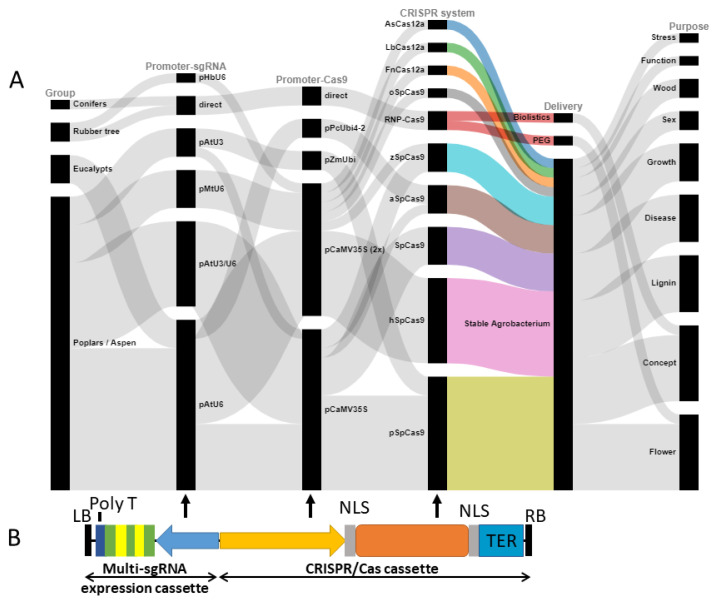
Overview of the constructed CRISPR/Cas systems that are used in genome editing of forest trees. (**A**) Alluvial diagram summarizing the background data from all 30 genome editing studies in forest trees. So far, CRISPR-mediated genome editing in forest trees has mostly been achieved in poplar and aspen species, mainly because the stable Agrobacterium-mediated transformation protocol is well established. Several different Poll III promoters of *Arabidopsis* (pAtU3/U6) or *Medicago* (pMtU6) and the CaMV35S promoter have been widely used to successfully produce singe/multiple sgRNA(s) and Cas nucleases, respectively, for genome editing in most of the reported tree species. However, it has been suggested that endogenous promoters pHbU6 may result in higher sgRNA expression in the rubber tree. For CRISPR-based genome editing in tree systems, several types of the engineered SpCas9 gene sequences with nuclear localization signals and designed with codon optimization for humans (hSpCas9), for *Arabidopsis thaliana* (aSpCas9), for maize (zSpCas9), for rice (oSpCas9), for plants with higher GC content at the 5′ terminal region (pSpCas9), or even the original coding sequence from *Streptococcus pyogenes* (SpCas9) have been successfully used. In addition, three Cas12a nucleases (i.e., AsCas12a, LbCas12a, and FnCas12a from *Acidaminococcus* spp., *Lachnospiraceae bacterium*, and *Francisella novicida*, respectively) were evaluated for the induction of targeted mutations in poplar. Besides the proof-of-concept and gene-function studies, genome editing efforts in trees focused on the incorporation of various silviculturally desirable traits including abiotic stress tolerance, wood quality, sex determination, growth enhancement, disease resistance, lignin modification, and flowering control. Detailed descriptions for each study can be found in Table S2. (**B**) A schematic diagram illustrating the typical T-DNA region of the constructed CRISPR/Cas vectors of which each sgRNA will be expressed by an individual promoter. There are several other systems to express multiple sgRNAs, such as using a tRNA backbone or a cys4 type of cleavage. LB, RB: Left and right borders; NLS: Nuclear localization signal; TER: Terminator sequence.

The successful implementation of the CRISPR system in tree species is still limited. The proof of concept for the CRISPR/Cas9 application has been established in several fruit tree species such as citrus [112,113], apple [114,115], grape [116], coffee [117], kiwifruit [118], cacao [119], pomegranate [120], walnut [121], and pear [115]. However, CRISPR-mediated genome editing in forest trees has been mainly achieved in poplar [122], and, for the last three years, in the tropical tree *Parasponia andersonii* [123], Eucalypts [124], rubber tree [125,126], Monterey pine [127], and European chestnut [128]. For evaluating CRISPR in new tree study systems, several types of the engineered SpCas9 gene sequences with nuclear localization signals and designed with codon optimization for human (hSpCas9, Addgene #42230 [11]), for *Arabidopsis thaliana* (aSpCas9, Addgene #61433, [129]), for rice (oSpCas9, Addgene #53064 [130]), for grasses including higher GC content at the 5′ terminal region (gSpCas9, Addgene #106331 [131]), or even the original coding sequence from *Streptococcus pyogenes* have been successfully used. The phytoene desaturase gene (PDS) is by far the most common endogenous target gene allowing for visual assessment of CRISPR/Cas9-induced knockout efficiency in trees because of the albino phenotype. There are only a few cases that improvements of CRISPR applications in the same or comparable tree systems have been observed, probably providing more specific suggestions for future CRISPR experiments in the systems for which there is still substantial room for optimization. For instance, the first report indicated successful knock-out of the phytoene desaturase (PDS) gene in the apple rootstock *Malus prunifolia* × *pumila* ‘JM2′ with edition rate of 31.8% [114]. In this case, the authors used the fungal and plant codon-optimized (GC-rich) version of the SpCas9 (called fcoCas9) fused to GFBSD2 (i.e., a GFP fused to the N-terminus of blasticidin S deaminase) under the control of the CaMV35S promoter. In addition, sgRNAs were separately under the control of the *A. thaliana* U6 promoter. The recent work demonstrated that a higher efficiency (84% [115]) of CRISPR/Cas9 editing in the apple PDS gene can be obtained by the simultaneous use of two sgRNAs driven by apple U3 and U6 promoters; and by using a simple SpCas9 with the *Arabidopsis* codon optimization [129], given that different studied genotypes could only partly explain the different rates of edition. Although *Arabidopsis* Pol III promoters and the CaMV35S promoter have been widely used to produce sgRNAs and Cas nucleases, respectively, for successful genome editing in most of the reported tree species (Figure 2, Supplemental Table S2), the initial attempt employing *Arabidopsis* and cotton U6 promoters for driving sgRNA transcription had failed to detect any edited plant in the rubber tree (*Hevea brasiliensis*). The CRISPR/Cas9 system could finally be established in *Hevea brasiliensis* by using any of five endogenous U6 promoters with a range of editing efficiencies from 8.47% to 24.92% [125]. Besides, directly compared with *Arabidopsis* promoters, species-specific U6 promoters were much more efficient for driving sgRNA expression and enhancing the editing efficiency of CRISPR/Cas9 systems in cotton [132] and soybean [133]. In another example, using the CaMV35S promoter to drive both hSpCas9 and sgRNA expression in sweet orange resulted in a relatively low frequency (3.2–3.9%) of CRISPR-induced mutations at the PDS locus [113]. The expression of hSpCas9 under the promoter of the *A. thaliana* YAO gene (which is preferentially expressed in the actively dividing tissues), using the same sgRNA increased the frequency of mutational events up to 75% in the citrus hybrid Carrizo Citrange [112], similar to the previous observation in *A. thaliana* [134]. This finding signifies room for improving the efficacy of CRISPR- mediated genome editing by optimizing expression patterns of CRISPR reagents.

## 6. Development of Highly Efficient and Precision Genome Editing Systems for Tree Species

The so-called CRISPR toolbox has expanded considerably to become optimized and advanced concerning specificity and efficiency (for review, see [89,92,135]). To overcome the limited target efficiency of SpCas9 due to the distribution of the specific PAM sequences in the target genome, alternative CRISPR/Cas systems using the Cas9 orthologues that were derived from other bacteria, such as *Staphylococcus aureus* (SaCas9, [136]), *Streptococcus thermophilus* (StCas9, [137]), and *Neisseria meningitides* (NmCas9, [138]), have also been developed for genome editing. For example, SaCas9 from *S. aureus*, is considerably smaller and recognizes a distinct 5′-NNGRRT protospacer adjacent motif (PAM) sequence (versus 5′-NGG of SpCas9), increasing the number of potential target sites of sgRNAs, especially in AT-rich regions which may facilitate promoter editing [139]. Jia et al. [140] effectively generated mutations in the Duncan grapefruit (*Citrus paradisi*) and Carrizo citrange (*Citrus aurantium*) by using the SaCas9 to successfully modify different target genes. The gene mutation efficiency was between 15.55% and 79.67%.

The CRISPR/Cas12a (Cpf1, classified as the class 2/type V) system has recently become a popular CRISPR effector, in addition to the conventional CRISPR/Cas9 Type II, presenting an advanced, simplified, and more efficient approach for genome editing [141,142]. Particularly, Cas12a differs from Cas9 as follows: (1) Cas12a recognizes the T-rich PAM sequence (e.g., 5′-TTTV-3′, targeting new genomic locations); (2) Cas12a cleavages with 5′ overhangs; (3) Cas12a is smaller in size and guided by a shorter crRNA (i.e., ~43–60 nucleotides, allowing a chemically synthesized crRNA that is more suitable for multiplexed editing and packing into viral vectors); and (4) there is a long distance between the recognition sequence and the cleavage site, promoting large chromosomal deletions and homology-dependent repair, or enabling reengineering at the same region (while genome editing by other CRISPR effectors including Cas9 causes the loss of a target site after the first-time engineering). The Cas12 orthologues from *Acidaminococcus* spp. (AsCas12a), *Francisella novicida* (FnCas12a), and *Lachnospiraceae bacterium* (LbCas12a) have been used to edit several plant models, such as rice [143,144], soybean [145], tobacco [146], tomato [147], and maize [148]. In the first application of the CRISPR/Cas12a system to woody plants [149], the LbCas12a system was used to successfully modify the Duncan grapefruit genome using either the transient expression of LbCas12a via Xcc-facilitated agroinfiltration or the constitutive expression of LbCas12a in transgenic plants. Interestingly, modification of the PthA4 effector binding elements in Type I CsLOB1 promoter (in total two alleles, Type I and Type II, of CsLOB1 in Duncan grapefruit) using specific Cas9/sgRNA-produced transgenic Duncan grapefruit plants alleviated *Xanthomonas* infection [150]. The activation of a single allele (the Type II which was not mutated by the Cas9/sgRNA and no suitable Cas9/sgRNA can be designed for both alleles) of the susceptibility gene CsLOB1 is, however, sufficient to induce citrus canker disease. Mutations in the promoters of both alleles of CsLOB1 were achieved by a single Cas12a/crRNA targeting a conserved region of both alleles [149], suggesting CRISPR/Cas12a as a versatile complementary tool for heterozygous genome editing, in addition to CRISPR/SpCas9 and SaCas9. Recently, three Cas12a nucleases (i.e., AsCas12a, LbCas12a, and FnCas12a) which were codon-optimized for rice [151], were evaluated for the induction of targeted mutations of the PDS gene in poplar (*Populus alba* × *Populus glandulosa*, [152]). In the poplar system, AsCas12a was the most efficient CRISPR system with the highest mutation efficiency of 70%, while LbCas12a performed better in rice [151,153]. These results demonstrate that the genome editing efficiency of CRISPR-effector variants needs to be tested in each specific organism. Especially, it would be worth testing whether other CRISPR/Cas12a variants that have recently been discovered and newly developed in rice [154,155] could be harnessed to efficiently generate genome-modified trees. In general, the Cas12a-induced mutations were mainly large deletions in the biallelic, non-mosaic state, suggesting a highly suitable tool for genome editing in forest trees for which the self-pollination practice for screening of the desired homozygous progeny is often very difficult (i.e., due to the time delay to onset of flowering, or intolerance of inbreeding [156]).

Several new natural CRISPR/Cas effectors have recently been discovered that could potentially be applied for genome editing in forest trees. For example, a small-sized CRISPR/Cas9 orthologue (~984 aa, [157]) from *Campylobacter jejuni* (CjCas9) and a set of CasX (likely classified into CRISPR/Cas12e type V, ~980 aa, [158]) were demonstrated as promising genome-editing tools (compared with 1368 aa of the commonly used SpCas9, or ~1200 aa of so far reported Cas12), offering possible advantages in increasing the delivery efficiency of CRISPR reagents that is a common obstacle in genome editing of many tree species. Furthermore, a new RGEN family of the CRISPR/Cas14 system (similar to the type V) from uncultivated archaea has an exceptionally compact size (400–700 aa), and the ability to target single-stranded DNA efficiently without the requirement of a PAM-sequence [159]. Such a PAM-free or a near PAM-free CRISPR system can unlimitedly expand the targetable chromosomal space in genome editing [160,161]. Finally, a unique genome editing tool from the Class 1 CRISPR/Cas3 (Type I-E) quickly and accurately triggered large deletions, up to 424 kb, upstream of a target site [162,163]. This unique characteristic would be useful for creating gene knockouts in trees by causing full-length gene deletions, while CRISPR/Cas9-mediated gene knockouts with small indels frequently produce truncated proteins. Besides, targeted large genomic deletions by CRISPR/Cas3 will facilitate the manipulation of repetitive and non-coding regions, having a broad impact on genome research in forest tree species that have an enormous proportion of repetitive sequences in the genome. Recently, the Type I-E CRISPR/Cascade system from *Streptococcus thermophilus* (StCascade) has been adopted for DNA targeting in *Zea mays* (Addgene #132334–132353, [164]) and repurposed for gene activation with greater effects than the CRISPR/Cas9 system. While the simple 5′ PAM (i.e., A or AA for StCascade) of the Type I-E CRISPR/Cascade system expands the potential targets in the genome, the long sgRNA target recognition sequences (~30–44 nucleotides) increase the specificity of DNA target identification. In general, the system provides great potential to advance genome editing. For instance, the DNA nuclease domain of Cas3 can be associated with the Cascades for large targeted chromosomal deletions or knock-in modifications by HDR, offering better opportunities for removing footprints of transgenic constructs, restructuring plant chromosomes, rearrangement of linkage groups, and overcoming hurdles in the fields of tree breeding or forest management.

In the CRISPR-based genome editing, transgene integration by HDR often remains challenging, partly due to the pre-dominance of the NHEJ repair pathway and the insufficient availability of repair templates at the site of the DSBs [165,166,167]. In animal and plant models, different approaches have been used to enhance HDR by regulating the cell cycle (i.e., animal cells are synchronized in S/G2 phases), chemically or genetically inhibiting genes that are involved in NHEJ (for review, see [167,168]). For instance, in human and mouse cell lines, the suppression (i.e., by gene silencing, small-molecule inhibition, or proteolytic degradation) of NHEJ key players DNA ligase IV, KU70, or KU80 is an effective way for engineering precisely targeted mutations into the genome [169]. Although the suppression by RNA interference of Ku70/80 or DNA ligase IV in rice calli also enhanced homologous recombination frequency, it decreased Agrobacterium-mediated stable transformation [170]. In many plant systems, including tree species, Agrobacterium-mediated transformation is the most practical means of transformation because of longer and more intact DNA payloads with less incorporation of fragmented DNA. In addition, provided that the HDR components are mainly active in the late S and G2 phase of the cell cycle, Cas9 driven by the egg cell- or early embryo-specific DD45 gene promoter achieved a promising frequency of inheritable gene replacements [171,172]. The first report for CRISPR-mediated gene replacement in tree models, poplar, was performed by simultaneous inhibition of NHEJ recombination cofactor XRCC4 and overexpression of HDR enhancer factors CtlP and MRE11 [173]. Importantly, not only the HDR-mediated knock-in efficiency was up to 40-fold greater, but also the products with the CRISPR-induced Indels, as outcomes of NHEJ repair mechanism, were seven-fold fewer, resulting in no functional effects on the gene nearby the target site. Nevertheless, HDR is a valuable and flexible tool for tree breeding applications that require precise knock-in of long DNA sequences/genes and complex DNA modifications. Recently, substantial advancements have been made in increasing the efficiency of HDR-mediated editing by different approaches, such as tandem repeat-HDR (TR-HDR, [174]) and transcript-templated HDR (TT-HDR, [175]). For example, by using chemical DNA modification of the donor DNA, Lu et al. inserted sequences including enhancers and promoters up to 2 kbp into the rice genome at an average efficiency of 25% [174]. The method is particularly useful for the precise insertion of regulatory elements to simultaneously manipulate the expression levels of multiple genes of interest.

The recent invention of CRISPR-mediated base editing and prime editing has opened new avenues for plant genome editing without donor DNA and a DSB introduction in the genome. Firstly, a cytosine base editor, the fusion of a nickase CRISPR/Cas9 and a cytidine deaminase enzyme, has enabled targeted conversions of cytosine to thymine [176,177]. Recent advancements in the base editing toolbox are indeed a leap forward in precise DNA base substitutions, including A–G base transition [178], C–A transversion [179], and C–G transversion [179,180,181]. In comparison with CRISPR-mediated HDR, base editing approaches can exhibit about 10 to 100-fold higher efficiency (for a review, see [182]). Secondly, a newly developed “search-and-replace” genome-editing technique is referred to as the prime editing using a fusion between nickase CRISPR/Cas9 and reverse transcriptase [183]. Importantly, the prime-editing guide RNA (pegRNA) is a guide RNA that also encodes the reverse-transcription template, which includes the desired edits (i.e., small up-to-44-bp insertions, up-to-80-bp deletions, and all 12 possible base-to-base conversions) and homology to the genomic DNA locus. Besides, the prime editor can edit near or far from PAM sites making it less constrained by PAM availability in the target genome in the same way as other CRISPR-based methods. Overall, these new precise nucleotide-editing strategies could further expand the CRISPR-based applications for the development of novel quantitative traits with a gain-of-function mutation [184,185]. In addition to its numerous advantages over conventional CRISPR-based systems, there are still certain aspects (e.g., on-target editing efficiency, unwanted mutations, optimal experiment design [186]) that need to be improved further for a more efficient and robust genome editing application.

## 7. Future Challenges and Concluding Remarks

The negative effects of climate change and climate variability on forest health are evident around the world. These impacts, such as the increasing intensity, frequency, and severity of heat waves, droughts, storms as well as pest and disease outbreaks are likely to be unavoidable, forcing trees to cope, adapt, or die. However, there is still much to learn about the mechanistic and ecological understanding of physiological adjustments and adaption of forest trees. As the number of reference genomes and the amount of genomic resources for forest tree species increase, the genetic basis of tree adaptation to new environmental conditions can be identified in a faster and higher resolution by using advanced genomic-assisted approaches (for review, see [187]), whole-genome resequencing, and pan-genome sequencing projects [51,188] as well as by CRISPR-enabled functional genomic studies (for review, see [23,189]). For example, by exploiting the recently available reference genome, two quantitative trait loci (QTL) that are associated with *Erysiphe alphitoides* infection were found in the pedunculate oak (*Q. robur*) genome regions [190] containing receptor-like-kinases and galactinol synthases as candidate genes. Besides, key components of temperature-mediated control of bud break have recently been discovered in aspen [191] and poplar [192], enabling approaches to modify dormancy-associated traits in temperate and boreal trees. Trees with better synchronization of bud phenology with local climate can avoid significant damage from early and late frosts, the outbreak of pests, and disease problems.

The availability of high-quality reference genomes of the target species is one of the prerequisites for confidence and comparability in genome editing assessment. The sequences are used for optimizing sgRNA design with the concerns of specificity, potential off-target products, local allelic variants affecting the efficiency, and genome context and available DNA machinery determining the editing outcomes. If there are only high-quality reference genome sequences of one or more closely related species, additional sequencing efforts need to be invested, including whole-genome resequencing, targeted amplicon sequencing, or a combination of multiple cloning and Sanger sequencing reactions. Up to now, among approximately 60,000 tree species and more than 100 chromosome-level reference genomes are available. Another 100 high-quality chromosome-level genomes are expected to be delivered in a couple of years, given the current development and advanced progress in long-read sequencing and long-range mapping technologies. Although in many cases gene annotation may be inferable from the presence of conserved sequence signatures, the identification of the precise biological role of genes, networks, and metabolic pathways, especially taxon-specific gene families, requires intensive experimental analysis on gene functional characterization. Classical genetic manipulation, which was a critical feature of established plant models, may no longer be essential thanks to the availability of rapid whole-genome sequencing and targeted gene editing by CRISPR technology. The expansion of comprehensive information on the annotated genomes of forest trees will present a substantial opportunity for tree improvement.

Since most forest tree species are either largely undomesticated or in the very early stages of domestication, the high genetic diversity in native tree populations could provide useful resources for tree breeding, “plus tree” selection, as well as guidance for highly effective genome editing strategies. Together with conventional breeding and transgenic approaches, precise and multiplex CRISPR-based genome editing tools greatly enhance opportunities for tree improvement in environmental adaptability and productivity, given that the majority of causative genes for important traits are uncovered. The traits for tree improvement include flowering traits, wood quality, cell wall modification, lignin content, photoperiodism, sterility, branching form, sex determination, hormone signaling, disease resistance, to name a few. For instance, introduced pests (e.g., emerald ash borer (*Agrilus planipennis*), southern pine beetle (*Dendroctonus frontalis*), gypsy moth (*Lymantria dispar*), sudden oak death (*Phytophthora ramorum*), and fusiform rust (*Cronartium quercuum* f. sp. *fusiform.*)) are killing or damaging millions of hectares of conifers and angiosperm trees each year. As a result, the long-term survival of many forest species (e.g., American chestnut, American ash species, European ash) is threatened [193,194]. To introduce resistance, for example, from Chinese chestnut into American chestnut, traditional breeding that requires many generations of back crosses has not been successful after several decades [195]. Importantly, many traits such as disease tolerance and abiotic stress resistance are controlled by a quite large number of QTL/genes with small effects on the phenotype [196,197]. Given that those difficulties are key challenges for tree breeding in general, CRISPR-based genome editing holds tremendous potential for the improvement of disease/pathogen resistance for rescuing forest trees (for a review and proposed applications, see [198,199]) together with other climate-resilient traits (e.g., for engineering drought resistance, see [197]). To take a complementary approach to traditional breeding, many plans for gene editing to restore the American chestnut were proposed [200]. So far, the genome editing method has been especially applicable for traits that are controlled by a relatively low number of genes (i.e., fewer than 10). However, it is still a complicated task for CRISPR-driven improvement of traits that are highly polygenic and regulated by complex genetic networks. As a result, the introduction of genomic changes can create imbalances in the network with unintended consequences or can produce different outcomes among different genetic backgrounds. This limitation might be alleviated by sequential editing or by pyramiding beneficial CRISPRed alleles through genetic crosses and marker-assisted selection.

Before realizing their full potential, these emerging genome editing technologies, including CRISPR-mediated HDR, base editing, and prime editing, are still under rapid evolution for improved efficiency, enhanced specificity and capability, and refined editing simplicity. Nevertheless, we anticipate that CRISPR-based technologies can contribute to studies of adaptive and climate-resilient traits in forest trees in the three aspects: testing candidate gene function, validation and quantifying the effect of allelic variants, and direct evolution of novel adaptive variations. Firstly, high-throughput and high-efficient CRISPR-based gene editing platforms that have been established in several CRISPR-compatible tree models can be used to reveal/validate gene functions (e.g., by simple knockout or loss-of-function mutations). Targets can be defined either from genome-wide association studies, genomic (QTL) synteny analysis of closely related tree species or from successful gene-editing studies of plant models (for a list of candidate genes for enhancing the abiotic stress tolerance of plants, see [201]). Secondly, once adopted to forest trees, precise knock-in CRISPR systems especially with gene replacement, base editing, and prime editing can introduce in-frame variations of protein-coding genes. In that way, a fraction of single nucleotide polymorphisms (SNPs) that are associated with stress-tolerance traits can be modified, resulting in plants with heritable and beneficial mutations. Besides, highly deleterious or climate-sensitive alleles can be precisely corrected or removed in breeding populations. Furthermore, given that regulatory elements such as enhancers and promoters can be simultaneously inserted or exchanged in multiple genes of interest, fine-tuning the expression levels of a desirable gene network/pathway while leaving the rest of the genome unaltered becomes feasible. Thus, CRISPR tools can be particularly useful for the improvement of quantitative traits. Thirdly, CRISPR can introduce novel variations, allowing a gain-of-function for the gene of interest. It is particularly important for the breeding of disease and pathogen resistance where the natural variation seems very limited. For example, de novo herbicide resistance mutations of the rice acetyl coenzyme A carboxylase (OsACC) gene can be generated from a range of near-saturated mutagenesis by using a CRISPR system with dual-base editors (i.e., introducing simultaneous A–G and C–G mutations) [202], or by using a prime-editing system with a comprehensive pegRNA library [203]. However, because under natural conditions trees are periodically or temporally exposed to combinations of stresses, the positive effect that is gained by a single genome editing may be overruled. Therefore, there is a strong need for long-term characterization studies of gene-edited trees in their natural environments.

To fully realize the potential of CRISPR-mediated gene editing in forestry, more methodological breakthroughs in the CRISPR technology are needed, especially on the, as yet inefficient, delivery of CRISPR reagents and the dependency on tissue culture. The majority of stable gene-edited trees are produced through tissue culture, where CRISPR reagents are delivered to sterile explants, and then the edited cells are regenerated into whole plants. It is routine in many herbaceous plants to self-pollinate and then screen progeny for a combination of desired edits and the absence of the CRISPR editing machinery. However, it is very time-consuming and problematic to do so in trees, often due to the delay in reproduction, intolerance of inbreeding, sterility, and loss of integrity after sexual segregation. If the CRISPR/Cas and gRNA genes must be removed from the edited lines due to regulatory needs or biological concerns, methods for editing without integrated transgenes, or technologies for removing integrated transgenes, will be required. When taking into account the removal of CRISPR functional components, recombinase excision approaches have been validated in several tree species, including poplars [204,205] and apple [206]. A drawback of this strategy is that a small residual “footprint” from the original T-DNA insertion will still be present in the genome, requiring more improvement of the technique. For CRISPR editing without integration, transient DNA delivery or viral delivery approaches can be considered. The most common technique for transient and physical DNA delivery in animal systems is using the RGEN and the associated sgRNA as a pre-packaged ribonucleic protein complex (RNP). In plants, the use of DNA-free physical transformation is commonly employed with cultured protoplasts, to then regenerate the protoplasts into intact plantlets in vitro [207]. Efficient genome editing of protoplasts from the rubber tree [126] and Dahurian larch (a coniferous tree [161]) has been demonstrated; however, the protoplast regeneration systems are very challenging in trees in general, and particularly in the transformed protoplasts. Alternative transformation methods can also be considered to deliver RNPs while bypassing the regeneration steps, including de novo meristem induction [208]. Besides, genome editing that is mediated by direct delivery (i.e., by particle bombardment) of Cas9 RNP has recently been applied to edit the gene for glucuronic acid substitution of xylan 1 (GUX1) in *Pinus radiata*, the most extensively planted exotic conifer species [127]. Using the RNP approach, somatic embryogenic cells were successfully mutated at the target site (with 22–33% efficiency), however, producing only monoallelic plantlets. The approach needs to be further optimized to increase the frequency of biallelic edits. To avoid tissue culture at which unintended mutations may also occur, attempts have been made to achieve CRISPR genome editing by using viral delivery systems. The main stumbling block of this strategy is the modest gene delivery payload of the virus system (i.e., the tobacco mosaic virus is typically <1 kb), precluding their use for delivering SpCas9 reagents (~4.1 kb). More recently advanced strategies may expand the versatility of these systems. For instance, the Sonchus yellow net virus (SYNV, [209]) and the potato virus X (PVX, [210]) were shown to be capable of delivering both Cas9 and gRNAs throughout tobacco plants. Given that the host range of SYNV and PVX is limited, an extensive search for viruses with similar cargo capacity but that are broadly compatible is needed. The optimization of CRISPR-based genome editing protocols to achieve transgene-free trees will facilitate the rapid deployment where DNA-free editing is not regulated as a GMO (for current regulations of CRISPR-edited plants, read reviews [156,211,212]). Furthermore, in the case where gene flow and seed/pollen dispersal from CRISPR-edited plantation plants to natural populations need to be prevented, full sterile trees (e.g., by knock-out the floral homeotic gene AGAMOUS (AG) and its close homologues [213,214,215]) with desired traits can be vegetatively propagated by the forest or horticulture industries.

Even though the biological concerns over possible gene drive are negligible, more attention should be paid to the detection of off-target mutations in trees due to the long generation time and preponderance of out-crossing in wind-pollinated species, such as oaks [216]. Evidence for extremely low rates of off-target mutations as well as measuring outcomes of CRISPR-mediated on-target damage in trees [15] has typically been assayed by using 1kb-range methods such as Sanger or short-read amplicon sequencing. Recent reports in animal models using long-read sequencing demonstrated that unexpected on-target damage of CRISPR was far more serious and widespread than anticipated [217,218] and revealed unforeseen CRISPR-Cas9 off-target activity [219,220]. Recently, high-throughput whole-genome resequencing has been used to evaluate off-target edits and untargeted mutations in *Arabidopsis* [221], rice [222,223], tomato [224], cotton [225], and grapevine [226], confirming that the off-target CRISPR-Cas9-induced mutations are rare in plants and smaller in magnitude than the variation that is generated by conventional tissue culturing or mutation breeding [227]. However, one possible limitation of the whole-genome resequencing approach for screening a large number of potential off-target sites is that it requires a reference genome.

In conclusion, CRISPR technology is a unique method with great potential for precise genome editing in forest trees. Studies that are summarized in this review represent only the first steps in the era of smart forests. CRISPR-mediated improvement (e.g., by editing a low number of genes) of wood quality, resistance to viruses, herbicides, drought, salt, and cold has already been reported in several tree models [15,197,228]. Moreover, the robustness of the CRISPR technology enables scientists to deploy newly developed and optimized systems from other plant models (such as CRISPR-mediated genome editing projects for developing climate-resilient crops and fruit trees) in forest tree breeding, climate resilience reforestation, forest health, and conservation. We highlighted here several advanced CRISPR systems as well as novel strategies for overcoming current large obstacles in forest tree systems, bearing the potential to be applicable in all forest tree species. With this significant progress in sequencing and CRISPR technologies within sight, a new green revolution in forestry might become reality in time.

## Data Availability

Detailed data of all figures are provided in Supplementary Materials.

## Supplementary Materials

The following are available online at https://www.mdpi.com/article/10.3390/ijms23020966/s1
