# Supplementary material for: From Genome Sequencing to CRISPR-Based Genome Editing for Climate-Resilient Forest Trees
Source: Int J Mol Sci. 2022 Jan 16;23(2):966. doi: 10.3390/ijms23020966 (PMC8780650; doi:10.3390/ijms23020966)
Supplement: Supplementary file 1 [file ijms-23-00966-s001.zip › Table S1.pdf]

Supplemental Data S1: Detailed data of Figure 1: Published reference genomes of trees and woody plant species. * Chromosome-level genome assembly.

Species	Common name	Genome size (Mbp)	Publication Year(s)	Nr genome version	Nr Reference Genotype	Resequencing Genotypes	Chromosome*	Reference
**Gymnospermae**								
Cupressaceae								
Sequoiadendron giganteum	Giant sequoia	8,125	2020	1	1	0	Yes	[1]
Taxaceae								
Taxus chinensis	Chinese yew	10,230	2021	1	1	0	Yes	[2]
Taxus wallichiana	Himalayan yew	10,900	2021	1	1	0	Yes	[3]
Pinaceae								
Picea abies	Norway spruce	19,700	2013	1	1	0		[4]
Picea glauca	White spruce	20,800	2013,-5	4	2	0		[5, 6]
Pinus taeda	Loblolly pine	21,600	2014,-7	2	1	0		[7-9]
Pinus lambertiana	Sugar pine	31,000	2016	1	1	0		[10]
Pseudotsuga menziesii	Douglas-fir	16,100	2017	1	1	0		[11]
Abies alba	Silver fir	17,360	2019	1	1	0		[12]
Larix sibirica	Siberian larch	12,340	2019	1	1	0		[13]
Ginkgoaceae								
Ginkgo biloba	Ginkgo tree	11,750	2016-21	2	1	0	Yes	[14, 15]
**Angiospermae**								
Piperaceae								
Piper nigrum	Black pepper	762	2019	1	1	0	Yes	[16]
Annonaceae								
Annona muricata	Soursop tree	800	2021	1	1	0	Yes	[17]
Magnoliaceae								
Liriodendron chinense	Tulip poplar	1,750	2019	1	1	20	Yes	[18]
Magnolia biondii	Biondi magnolia	2,240	2021	1	1	0	Yes	[19]
Magnolia officinalis	HouPo magnolia	1,760	2021	1	1	0	Yes	[20]
Calycanthaceae								
Chimonanthus praecox	Wintersweet	780	2020	1	1	0	Yes	[21]
Chimonanthus salicifolius		836	2020	1	1	0	Yes	[22]

Lauraceae								
Cinnamomum kanehirae	Stout camphor	830	2019	1	1	0	Yes	[23]
Litsea cubeba	May Chang tree	1,370	2020	1	1	47	Yes	[24]
Persea americana	Avocado	980	2019	1	1	10	Yes	[25]
Poaceae								
Bonia amplexicaulis	woody bamboo	848	2019	1	1	0		[26]
Guadua angustifolia	woody bamboo	1,614	2019	1	1	0		[26]
Arecaceae								
Areca catechu	Areca palm	2,590	2021	1	1	0	Yes	[27]
Calamus simplicifolius	Danye rattan	1,980	2018	1	1	0	Yes	[28]
Cocos nucifera	Coconut palm	2,420	2017-20	1	3	0		[29-31]
Daemonorops jenkinsiana	Ginseng rattan	1,610	2018	1	1	0	Yes	[28]
Elaeis guineensis	African oil palm	1,800	2013-16	2	2	17	Yes	[32, 33]
Elaeis oleifera	Oil palm	1,800	2013	1	1	0		[32]
Korthalsia laciniosa	Climbing rattan	592	2021	1	1	0		[34]
Phoenix dactylifera	Date palm	772	2011-9	3	2	145		[35-37]
Proteaceae								
Macadamia integrifolia	Macadamia nut	650	2016-20	2	1	0	Yes	[38, 39]
Trochodendraceae								
Tetracentron sinense		1,120	2020-1	2	2	55	Yes	[40, 41]
Trochodendron aralioides	Wheel tree	1,758	2019	1	1	0	Yes	[42]
Santalaceae								
Santalum album	Sandalwood	220	2018	1	1	0		[43]
Olacaceae								
Malania oleifera	Garlic-fruit tree	1,500	2019	1	1	0		[44]
Actinidiaceae								
Actinidia chinensis	Golden kiwifruit	758	2013-9	3	2	0	Yes	[45-47]
Actinidia eriantha	Velvet kiwifruit	745	2019	1	1	0	Yes	[48]
Ericaceae								
Rhododendron delavayi	Tree rhododendron	700	2017	1	1	30		[49]
Rhododendron griersonianum		750	2021	1	1	31	Yes	[50]

Rhododendron ripense	Kishi azalea	527	2021	1	1	0	Yes	[51]
Rhododendron kiyosumense	Wild azalea	591	2021	1	1	0		[51]
Rhododendron ovatum	azalea	549	2021	1	1	0	Yes	[52]
Rhododendron simsii	Sim's azalea	530	2020	1	1	0	Yes	[53]
Rhododendron williamsianum	Williams rhododendron	651	2019	1	1	0	Yes	[54]
Vaccinium corymbosum	American blueberry	500	2015-9	1	2	0	Yes	[55]
Vaccinium macrocarpon	American cranberry	493	2014-21	1	2	0	Yes	[56, 57]
Vaccinium microcarpum	Small cranberry	490	2021	1	1	0		[57]
Vaccinium myrtillus	Bilberry	600	2021	1	1	0	Yes	[58]
Primulaceae								
Aegiceras corniculatum	Black mangrove	905	2021	1	1	0	Yes	[59]
Ebenaceae								
Diospyros lotus	Persimmon	907	2020	1	1	0	Yes	[60]
Diospyros oleifera	Oily persimmon	850	2019-20	1	2	0	Yes	[61, 62]
Sapotaceae								
Argania spinosa	Argane tree	573	2020	1	1	0		[63]
Theaceae								
Camellia sinensis	Tea tree	3,000	2017-21	2	7	190	Yes	[64-72]
Nyssaceae								
Camptotheca acuminata	Happy tree	516	2017-21	2	1	0	Yes	[73, 74]
Davidia involucrata	Dove tree	1,169	2020	1	1	10	Yes	[75]
Nyssa sinensis	Chinese tupelo	1,000	2019	1	1	0	Yes	[76]
Nyssa yunnanensis	Yunnan tupelo	1,640	2020	1	1	0		[77]
Paulowniaceae								
Paulownia fortunei	Fortune's empress tree	540	2021	1	1	0	Yes	[78]
Acanthaceae								
Avicennia marina	Gray mangrove	460	2021	1	2	0	Yes	[79, 80]
Oleaceae								
Forsythia suspensa	Weeping forsythia	701	2020	1	1	300		[81]

Fraxinus excelsior	European ash	880	2017	5	1	37		[82]
Olea europaea	Olive tree	1,380	2016-21	3	3	0	Yes	[83-85]
Osmanthus fragrans	Sweet osmanthus	730	2019-21	2	2	122	Yes	[86, 87]
Bignoniaceae								
Handroanthus guayacan		510	2021	1	1	0		[88]
Handroanthus impetiginosus	Pink trumpet	557	2018	1	1	0		[89]
Jacaranda copaia		800	2021	1	1	0		[88]
Jacaranda mimosifolia	Blue jacaranda	740	2021	1	1	0	Yes	[90]
Lamiaceae								
Tectona grandis	Teak	465	2018-9	2	2	0	Yes	[91, 92]
Solanaceae								
Lycium barbarum	Goji berry	1,800	2021	1	1	12	Yes	[93]
Rubiaceae								
Coffea arabica	Arabian coffee	1,300	2018-20	2	2	787		[94]
Coffea canephora	African coffee	710	2014	1	1	35	Yes	[95]
Coffea humblotiana	Wild coffee	422	2021	1	1	0	Yes	[96]
Mitragyna speciosa	Kratom	780	2021	1	1	0		[97]
Eucommiaceae								
Eucommia ulmoides	Hardy rubber	1,200	2018-20	2	2	0	Yes	[98, 99]
Vitaceae								
Vitis amurensis	Amur river grape	610	2021	1	1	0	Yes	[100]
Vitis riparia	Riverbank grape	495	2019-20	2	2	0	Yes	[101, 102]
Vitis vinifera	Grapevine	475	2007-20	1	3	472	Yes	[103-106]
Muscadinia rotundifolia	Muscadine grape	958	2020-1	2	1	0	Yes	[107]
Fabaceae								
Dalbergia odorifera	Fragrant rosewood	654	2020	1	1	0	Yes	[108]
Faidherbia albida	apple-ring acacia	661	2018	1	1	0		[109]
Rosaceae								
Cydonia oblonga	Quince	686	2021	1	1	0		[110]
Eriobotrya japonica	Loquat	711	2020-1	1	2	0	Yes	[111, 112]
Malus baccata	Siberian crab apple	778	2019	1	1	6	Yes	[113]

Malus x domestica	Apple	742	2010-20	1	3	43	Yes	[114-118]
Malus sieversii	Wild apple	668	2020	1	1	37	Yes	[118]
Malus sylvestris	Wild apple	661	2020	1	1	11	Yes	[118]
Prunus armeniaca	Apricot	230	2021	1	1	520	Yes	[119]
Prunus avium	Sweet cherry	353	2017-20	2	3	97	Yes	[120-123]
Prunus domestica	Common plum	1,400	2021	1	1	0		[124]
Prunus dulcis	Almond	240	2020	1	1	10	Yes	[125]
Prunus humilis		230	2020	1	1	0	Yes	[126]
Prunus mandshurica	Manchurian apricot	230	2021	1	1	0	Yes	[119]
Prunus mume	Chinese plum	280	2012	1	1	348	Yes	[127]
Prunus persica	Peach	265	2013-21	2	2	0	Yes	[128-130]
Prunus salicina	Japanese plum	310	2020-21	1	2	78	Yes	[131, 132]
Prunus serrulata	Asian cherry	257	2020	1	1	0	Yes	[133]
Prunus sibirica	Siberian apricot	225	2021	1	1	43	Yes	[119]
Prunus yedoensis	Yoshino cherry	690	2018-9	1	2	10	Yes	[134]
Moraceae								
Artocarpus altilis	Breadfruit	812	2019	1	1	0		[135]
Artocarpus camansi	Breadnut	669	2016	1	1	0		[136]
Artocarpus heterophyllus	Jackfruit	1,006	2019	1	1	0		[135]
Ficus carica	Common fig	356	2017	1	1	0		[137]
Ficus erecta	Japanese fig	340	2020	1	1	10	Yes	[138]
Ficus hispida	Hairy fig	370	2020	1	1	6	Yes	[139]
Ficus microcarpa	Laurel fig	436	2020	1	1	2	Yes	[139]
Salicaceae								
Populus alba	Silver poplar	536	2019	1	1	0		[140]
Populus alba x tremula	Hybrid poplar	748	2019	1	1	0		[141]
Populus euphratica	Euphrates poplar	593	2013	1	1	0		[142]
Populus alba x glandulosa	Hybrid poplar	782	2019-21	2	1	0		[143, 144]
Populus ilicifolia	African poplar	444	2020	1	1	18		[145]
Populus pruinosa	Desert poplar	590	2017	1	1	0		[146]
Populus tremula	European aspen	442	2018	1	1	24		[147]
Populus tremuloides	American aspen	560	2018	1	1	22		[147]

Populus trichocarpa	Black cottonwood	485	2006	1	1	24	Yes	[148]
Salix brachista	Cushion willow	420	2019	1	1	77	Yes	[149]
Salix dunnii		380	2021	1	1	38	Yes	[150]
Salix matsudana	Corkscrew willow	656	2020	1	1	0	Yes	[151]
Salix suchowensis	Shrub willow	425	2014-20	2	1	0	Yes	[152, 153]
Salix viminalis	Basket willow	360	2020	1	1	0		[154]
Euphorbiaceae								
Hevea brasiliensis	Rubber tree	2,150	2013-9	2	4	16	Yes	[155-159]
Jatropha curcas	Physic nut tree	380	2011-9	1	3	0	Yes	[160-162]
Vernicia fordii	Tung tree	1,200	2018-9	1	2	0	Yes	[163, 164]
Juglandaceae								
Carya cathayensis	Chinese hickory	721	2019	1	1	0		[165]
Carya illinoinensis	Pecan	650	2019-21	2	5	18	Yes	[165, 166]
Juglans cathayensis	Manchurian walnut	582	2018-21	1	2	0	Yes	[167, 168]
Juglans hindsii	Northern California walnut	577	2018	1	1	0		[167]
Juglans microcarpa	Texas black walnut	571	2018-9	1	2	0	Yes	[167, 169]
Juglans nigra	Eastern black walnut	583	2018	1	1	0		[167]
Juglans regia	Common walnut	606	2016-20	2	3	23	Yes	[167, 169, 170]
Juglans sigillata	Iron walnut	594	2018-20	1	2	0	Yes	[167, 171]
Pterocarya stenoptera	Chinese wingnut	600	2018	1	1	0		[167]
Betulaceae								
Alnus glutinosa	European alder	461	2018	1	1	0		[172]
Betula nana	Dwarf birch	450	2013	1	1	0		[173]
Betula pendula	Silver birch	440	2017	1	1	80	Yes	[174]
Betula platyphylla	Asian white birch	440	2021	1	1	0	Yes	[175]
Carpinus fangiana	Monkeytail hornbeam	397	2020	1	1	0	Yes	[176]
Corylus avellana	European hazelnut	378	2020-1	1	2	0	Yes	[177, 178]
Corylus heterophylla	Siberian hazelnut	374	2021	1	1	0	Yes	[179]

Corylus mandshurica	Manchurian hazelnut	392	2021	1	1	0	Yes	[180]
Ostrya chinensis	Ironwood tree	386	2018	1	1	13		[181]
Ostrya rehderiana	Ironwood tree	386	2018	1	1	13		[181]
Casuarinaceae								
Casuarina equisetifolia	Australian pine	300	2019	1	1	0		[182]
Casuarina glauca	Swamp oak	314	2018	1	1	0		[172]
Myricaceae								
Morella rubra	Red bayberry	320	2018-9	1	2	3	Yes	[183, 184]
Fagaceae								
Castanea crenata	Japanese chestnut	670	2021	1	1	0	Yes	[185]
Castanea mollissima	Chinese chestnut	800	2019	1	1	0		[186]
Fagus sylvatica	European Beech	541	2018	1	1	0		[187]
Quercus lobata	Valley oak	725	2016	1	1	0		[188]
Quercus robur	European oak	736	2018	1	1	0	Yes	[189]
Quercus suber	Cork oak	934	2018	1	1	0		[190]
Oxalidaceae								
Averrhoa carambola	Star fruit tree	475	2020	1	2	0	Yes	[191, 192]
Caricaceae								
Carica papaya	Papaya	372	2008	1	1	0	Yes	[193]
Moringaceae								
Moringa oleifera	Drumstick tree	315	2015-8	1	1	0		[109]
Malvaceae								
Bombax ceiba	Red silk-cotton tree	809	2018	1	1	0		[194]
Durio zibethinus	Durian	738	2017	1	1	0	Yes	[195]
Gossypium anomalum	Wild African cotton (B)	1,350	2021	1	1	4		[196]
Gossypium arboreum	Tree cotton (A2)	1,746	2014-20	2	1	67	Yes	[197, 198]
Gossypium australe	Wild cotton (G)	1,670	2020	1	1	0		[199]
Gossypium barbadense	Sea Island cotton (AD2)	2,470	2015-21	1	4	9	Yes	[200-203]

Gossypium darwinii	Darwin's cotton (AD5)	2,183	2020	1	1	0	Yes	[202]
Gossypium herbaceum	Levant cotton (A1)	1,560	2020	1	1	14	Yes	[198]
Gossypium hirsutum	Upland cotton (AD1)	2,340	2015-21	3	3	1081	Yes	[198, 201-204]
Gossypium longicalyx	Wild cotton F	1,311	2020	1	1	0	Yes	[205]
Gossypium mustelinum	Wild cotton AD4	2,315	2020	1	1	0	Yes	[202]
Gossypium raimondii	Wild cotton D	880	2012-9	1	2	0	Yes	[206, 207]
Gossypium tomentosum	Haiwaiian cotton AD3	2,194	2020-1	1	2	0	Yes	[202, 208]
Gossypium turneri	Wild cotton D	910	2019	1	1	0	Yes	[207]
Theobroma cacao	Cocoa	430	2011	1	1	0	Yes	[209]
Thymelaeaceae								
Aquilaria agallocha	Agarwood	736	2014	1	1	0		[210]
Aquilaria sinensis	Chinese agarwood	773	2020	1	2	0	Yes	[211, 212]
Staphyleaceae								
Euscaphis japonica	Korean sweetheart tree	1,390	2021	1	1	101	Yes	[213]
Tapisciaceae								
Tapiscia sinensis		410	2020	1	1	55		[214]
Myrtaceae								
Corymbia citriodora	Lemon-scented gum tree	380	2021	1	1	0	Yes	[215]
Eucalyptus camaldulensis	River red gum	655	2011	1	1	0		[216]
Eucalyptus grandis	Rose gum	640	2014	1	1	1	Yes	[217]
Eucalyptus pauciflora	Snow gum	530	2020	1	1	0		[218]
Melaleuca alternifolia	Tea tree	360	2021	1	1	0		[219]
Psidium guajava	Guava	464	2020	1	1	0	Yes	[220]
Lythraceae								
Punica granatum	Pomegranate	360	2017-20	1	3	26	Yes	[221-223]
Rutaceae								
Atalantia buxifolia	Atalantia	328	2017	1	1	15		[224]

Citrus clementina	Clementine mandarin	302	2014	1	1	27	Yes	[225]
Citrus grandis	Pummelo	381	2017	1	1	19		[224]
Citrus ichangensis	Ichang papeda	391	2017	1	1	12		[224]
Citrus medica	Citron	407	2017	1	1	9		[224]
Citrus paradisi x P. trifoliata	Swingle citrumelo	380	2016	1	1	0		[226]
Citrus reticulata	Wild mandarin	370	2018	1	1	13		[227]
Citrus sinensis	Sweet orange	367	2013	1	1	11	Yes	[228]
Citrus unshiu	Satsuma mandarin	370	2017	1	1	0	Yes	[229]
Poncirus trifoliata	Trifoliate orange	273	2020	1	1	5	Yes	[230]
Zanthoxylum bungeanum	Szechuan pepper	4,400	2021	1	1	0	Yes	[231]
Meliaceae								
Azadirachta indica	Neem tree	364	2012	1	1	0		[232]
Toona sinensis	Chinese mahogany	580	2021	1	1	0	Yes	[233]
Anacardiaceae								
Mangifera indica	Mango	360	2020-1	1	2	48	Yes	[234, 235]
Pistacia vera	Pistachio	520	2019	1	1	107		[236]
Sclerocarya birrea	Marula	356	2018	1	1	0		[109]
Sapindaceae								
Acer truncatum	Purpleblow maple	654	2020	1	1	0	Yes	[237]
Acer yangbiense		640	2019	1	1	0	Yes	[238]
Dimocarpus longan	Longan	480	2017	1	1	13		[239]
Nephelium lappaceum	Rambutan	340	2021	1	1	27	Yes	[240]
Xanthoceras sorbifolium	Yellowhorn	440	2019	1	2	0	Yes	[241, 242]

References:

1. Scott, A. D., A. V. Zimin, D. Puiu, R. Workman, M. Britton, S. Zaman, M. Caballero, A. C. Read, A. J. Bogdanove, E. Burns, *et al.* "A Reference Genome Sequence for Giant Sequoia." *G3 (Bethesda)* 10 (2020): 3907-19.
2. Xiong, X., J. Gou, Q. Liao, Y. Li, Q. Zhou, G. Bi, C. Li, R. Du, X. Wang, T. Sun, *et al.* "The Taxus genome provides insights into paclitaxel biosynthesis." *Nat Plants* 7 (2021): 1026-36.
3. Cheng, J., X. Wang, X. Liu, X. Zhu, Z. Li, H. Chu, Q. Wang, Q. Lou, B. Cai, Y. Yang, *et al.* "Chromosome-level genome of Himalayan yew provides insights into the origin and evolution of the paclitaxel biosynthetic pathway." *Mol Plant* 14 (2021): 1199-209.
4. Nystedt, B., N. R. Street, A. Wetterbom, A. Zuccolo, Y. C. Lin, D. G. Scofield, F. Vezzi, N. Delhomme, S. Giacomello, A. Alexeyenko, *et al.* "The Norway spruce genome sequence and conifer genome evolution." *Nature* 497 (2013): 579-84.
5. Warren, R. L., C. I. Keeling, M. M. Yuen, A. Raymond, G. A. Taylor, B. P. Vandervalk, H. Mohamadi, D. Paulino, R. Chiu, S. D. Jackman, *et al.* "Improved white spruce (*Picea glauca*) genome assemblies and annotation of large gene families of conifer terpenoid and phenolic defense metabolism." *Plant J* 83 (2015): 189-212.
6. Birol, I., A. Raymond, S. D. Jackman, S. Pleasance, R. Coope, G. A. Taylor, M. M. S. Yuen, C. I. Keeling, D. Brand, B. P. Vandervalk, *et al.* "Assembling the 20 Gb white spruce (*Picea glauca*) genome from whole-genome shotgun sequencing data." *Bioinformatics* 29 (2013): 1492-97.
7. Neale, D. B., J. L. Wegrzyn, K. A. Stevens, A. V. Zimin, D. Puiu, M. W. Crepeau, C. Cardeno, M. Koriabine, A. E. Holtz-Morris, J. D. Liechty, *et al.* "Decoding the massive genome of loblolly pine using haploid DNA and novel assembly strategies." *Genome Biol* 15 (2014): R59.
8. Zimin, A., K. A. Stevens, M. W. Crepeau, A. Holtz-Morris, M. Koriabine, G. Marcais, D. Puiu, M. Roberts, J. L. Wegrzyn, P. J. de Jong, *et al.* "Sequencing and assembly of the 22-gb loblolly pine genome." *Genetics* 196 (2014): 875-90.
9. Zimin, A. V., K. A. Stevens, M. W. Crepeau, D. Puiu, J. L. Wegrzyn, J. A. Yorke, C. H. Langley, D. B. Neale and S. L. Salzberg. "An improved assembly of the loblolly pine mega-genome using long-read single-molecule sequencing." *GigaScience* 6 (2017): 1-4.
10. Stevens, K. A., J. L. Wegrzyn, A. Zimin, D. Puiu, M. Crepeau, C. Cardeno, R. Paul, D. Gonzalez-Ibeas, M. Koriabine, A. E. Holtz-Morris, *et al.* "Sequence of the Sugar Pine Megagenome." *Genetics* 204 (2016): 1613-26.
11. Neale, D. B., P. E. McGuire, N. C. Wheeler, K. A. Stevens, M. W. Crepeau, C. Cardeno, A. V. Zimin, D. Puiu, G. M. Pertea, U. U. Sezen, *et al.* "The Douglas-Fir Genome Sequence Reveals Specialization of the Photosynthetic Apparatus in Pinaceae." *G3 (Bethesda)* 7 (2017): 3157-67.
12. Mosca, E., F. Cruz, J. Gomez-Garrido, L. Bianco, C. Rellstab, S. Brodbeck, K. Csillery, B. Fady, M. Fladung, B. Fussi, *et al.* "A Reference Genome Sequence for the European Silver Fir (*Abies alba* Mill.): A Community-Generated Genomic Resource." *G3 (Bethesda)* 9 (2019): 2039-49.
13. Kuzmin, D. A., S. I. Feranchuk, V. V. Sharov, A. N. Cybin, S. V. Makolov, Y. A. Putintseva, N. V. Oreshkova and K. V. Krutovsky. "Stepwise large genome assembly approach: a case of Siberian larch (*Larix sibirica* Ledeb)." *BMC Bioinformatics* 20 (2019): 37.
14. Liu, H., X. Wang, G. Wang, P. Cui, S. Wu, C. Ai, N. Hu, A. Li, B. He, X. Shao, *et al.* "The nearly complete genome of *Ginkgo biloba* illuminates gymnosperm evolution." *Nat Plants* 7 (2021): 748-56.
15. Guan, R., Y. Zhao, H. Zhang, G. Fan, X. Liu, W. Zhou, C. Shi, J. Wang, W. Liu, X. Liang, *et al.* "Draft genome of the living fossil *Ginkgo biloba*." *Gigascience* 5 (2016): 49.
16. Hu, L., Z. Xu, M. Wang, R. Fan, D. Yuan, B. Wu, H. Wu, X. Qin, L. Yan, L. Tan, *et al.* "The chromosome-scale reference genome of black pepper provides insight into piperine biosynthesis." *Nat Commun* 10 (2019): 4702.

17. Strijk, J. S., D. D. Hinsinger, M. M. Roeder, L. W. Chatrou, T. L. P. Couvreur, R. H. J. Erkens, H. Sauquet, M. D. Pirie, D. C. Thomas and K. Cao. "Chromosome-level reference genome of the soursop (*Annona muricata*): A new resource for Magnoliid research and tropical pomology." *Mol Ecol Resour* 21 (2021): 1608-19.
18. Chen, J., Z. Hao, X. Guang, C. Zhao, P. Wang, L. Xue, Q. Zhu, L. Yang, Y. Sheng, Y. Zhou*, et al.* "Liriodendron genome sheds light on angiosperm phylogeny and species-pair differentiation." *Nat Plants* 5 (2019): 18-25.
19. Dong, S., M. Liu, Y. Liu, F. Chen, T. Yang, L. Chen, X. Zhang, X. Guo, D. Fang, L. Li*, et al.* "The genome of *Magnolia biondii* Pamp. provides insights into the evolution of Magnoliales and biosynthesis of terpenoids." *Hortic Res* 8 (2021): 38.
20. Yin, Y., F. Peng, L. Zhou, X. Yin, J. Chen, H. Zhong, F. Hou, X. Xie, L. Wang, X. Shi*, et al.* "The chromosome-scale genome of *Magnolia officinalis* provides insight into the evolutionary position of magnoliids." *iScience* 24 (2021): 102997.
21. Shang, J., J. Tian, H. Cheng, Q. Yan, L. Li, A. Jamal, Z. Xu, L. Xiang, C. A. Saski, S. Jin*, et al.* "The chromosome-level wintersweet (*Chimonanthus praecox*) genome provides insights into floral scent biosynthesis and flowering in winter." *Genome Biol* 21 (2020): s13059-020-02088-y.
22. Lv, Q., J. Qiu, J. Liu, Z. Li, W. Zhang, Q. Wang, J. Fang, J. Pan, Z. Chen, W. Cheng*, et al.* "The *Chimonanthus salicifolius* genome provides insight into magnoliid evolution and flavonoid biosynthesis." *Plant J* 103 (2020): 1910-23.
23. Chaw, S. M., Y. C. Liu, Y. W. Wu, H. Y. Wang, C. I. Lin, C. S. Wu, H. M. Ke, L. Y. Chang, C. Y. Hsu, H. T. Yang*, et al.* "Stout camphor tree genome fills gaps in understanding of flowering plant genome evolution." *Nat Plants* 5 (2019): 63-73.
24. Chen, Y.-C., Z. Li, Y.-X. Zhao, M. Gao, J.-Y. Wang, K.-W. Liu, X. Wang, L.-W. Wu, Y.-L. Jiao, Z.-L. Xu*, et al.* "The Litsea genome and the evolution of the laurel family." *Nat Commun* 11 (2020): s41467-020-15493-5.
25. Rendon-Anaya, M., E. Ibarra-Laclette, A. Mendez-Bravo, T. Lan, C. Zheng, L. Carretero-Paulet, C. A. Perez-Torres, A. Chacon-Lopez, G. Hernandez-Guzman, T. H. Chang*, et al.* "The avocado genome informs deep angiosperm phylogeny, highlights introgressive hybridization, and reveals pathogen-influenced gene space adaptation." *P Natl Acad Sci USA* 116 (2019): 17081-89.
26. Guo, Z. H., P. F. Ma, G. Q. Yang, J. Y. Hu, Y. L. Liu, E. H. Xia, M. C. Zhong, L. Zhao, G. L. Sun, Y. X. Xu*, et al.* "Genome Sequences Provide Insights into the Reticulate Origin and Unique Traits of Woody Bamboos." *Mol Plant* 12 (2019): 1353-65.
27. Yang, Y., L. Huang, C. Xu, L. Qi, Z. Wu, J. Li, H. Chen, Y. Wu, T. Fu, H. Zhu*, et al.* "Chromosome-scale genome assembly of areca palm (*Areca catechu*)." *Mol Ecol Resour* (2021): 1755-0998.13446.
28. Zhao, H., S. Wang, J. Wang, C. Chen, S. Hao, L. Chen, B. Fei, K. Han, R. Li, C. Shi*, et al.* "The chromosome-level genome assemblies of two rattans (*Calamus simplicifolius* and *Daemonorops jenkinsiana*)." *Gigascience* 7 (2018): giy097.
29. Lantican, D. V., S. R. Strickler, A. O. Canama, R. R. Gardoce, L. A. Mueller and H. F. Galvez. "De Novo Genome Sequence Assembly of Dwarf Coconut (*Cocos nucifera* L. 'Catigan Green Dwarf') Provides Insights into Genomic Variation Between Coconut Types and Related Palm Species." *G3 (Bethesda)* 9 (2019): 2377-93.
30. Muliyar, R. K., P. Chowdappa, S. K. Behera, S. Kasaragod, K. P. Gangaraj, C. N. Kotimoole, B. Nekrakalaya, V. Mohanty, R. B. Sampgod, G. Banerjee*, et al.* "Assembly and Annotation of the Nuclear and Organellar Genomes of a Dwarf Coconut (Chowghat Green Dwarf) Possessing Enhanced Disease Resistance." *OMICS: A Journal of Integrative Biology* 24 (2020): 726-42.
31. Xiao, Y., P. Xu, H. Fan, L. Baudouin, W. Xia, S. Bocs, J. Xu, Q. Li, A. Guo, L. Zhou*, et al.* "The genome draft of coconut (*Cocos nucifera*)." *Gigascience* 6 (2017): 1-11.
32. Singh, R., M. Ong-Abdullah, E. T. Low, M. A. Manaf, R. Rosli, R. Nookiah, L. C. Ooi, S. E. Ooi, K. L. Chan, M. A. Halim, *et al.* "Oil palm genome sequence reveals divergence of interfertile species in Old and New worlds." *Nature* 500 (2013): 335-9.

33. Jin, J., M. Lee, B. Bai, Y. Sun, J. Qu, Rahmadsyah, Y. Alfiko, C. H. Lim, A. Suwanto, M. Sugiharti, *et al.* "Draft genome sequence of an elite Dura palm and whole-genome patterns of DNA variation in oil palm." *DNA Res* 23 (2016): 527-33.
34. Ghosh Dasgupta, M., S. A. Dev, A. B. Muneera Parveen, P. Sarath and V. B. Sreekumar. "Draft genome of *Korthalsia laciniosa* (Griff.) Mart., a climbing rattan elucidates its phylogenetic position." *Genomics* 113 (2021): 2010-22.
35. Al-Dous, E. K., B. George, M. E. Al-Mahmoud, M. Y. Al-Jaber, H. Wang, Y. M. Salameh, E. K. Al-Azwani, S. Chaluvadi, A. C. Pontaroli, J. DeBarry, *et al.* "De novo genome sequencing and comparative genomics of date palm (*Phoenix dactylifera*)." *Nat Biotechnol* 29 (2011): 521-7.
36. Hazzouri, K. M., M. Gros-Balthazard, J. M. Flowers, D. Copetti, A. Lemansour, M. Lebrun, K. Masmoudi, S. Ferrand, M. I. Dhar, Z. A. Fresquez, *et al.* "Genome-wide association mapping of date palm fruit traits." *Nat Commun* 10 (2019): 4680.
37. Al-Mssallem, I. S., S. Hu, X. Zhang, Q. Lin, W. Liu, J. Tan, X. Yu, J. Liu, L. Pan, T. Zhang, *et al.* "Genome sequence of the date palm *Phoenix dactylifera* L." *Nat Commun* 4 (2013): ncomms3274.
38. Nock, C. J., A. Baten, B. J. Barkla, A. Furtado, R. J. Henry and G. J. King. "Genome and transcriptome sequencing characterises the gene space of *Macadamia integrifolia* (Proteaceae)." *BMC Genomics* 17 (2016): 937.
39. Nock, C. J., A. Baten, R. Mauleon, K. S. Langdon, B. Topp, C. Hardner, A. Furtado, R. J. Henry and G. J. King. "Chromosome-Scale Assembly and Annotation of the Macadamia Genome (*Macadamia integrifolia* HAES 741)." *G3 (Bethesda)* 10 (2020): 3497-504.
40. Li, M., Y. Yang, R. Xu, W. Mu, Y. Li, X. Mao, Z. Zheng, H. Bi, G. Hao, X. Li, *et al.* "A chromosome-level genome assembly for the tertiary relict plant *Tetracentron sinense* oliv. (trochodendraceae)." *Mol Ecol Resour* 21 (2021): 1186-99.
41. Liu, P. L., X. Zhang, J. F. Mao, Y. M. Hong, R. G. Zhang, Y. E, S. Nie, K. Jia, C. K. Jiang, J. He, *et al.* "The Tetracentron genome provides insight into the early evolution of eudicots and the formation of vessel elements." *Genome Biol* 21 (2020): 291.
42. Strijk, J. S., D. D. Hinsinger, F. Zhang and K. Cao. "*Trochodendron aralioides*, the first chromosome-level draft genome in Trochodendrales and a valuable resource for basal eudicot research." *Gigascience* 8 (2019): giz136.
43. Mahesh, H. B., P. Subba, J. Advani, M. D. Shirke, R. M. Loganathan, S. L. Chandana, S. Shilpa, O. Chatterjee, S. M. Pinto, T. S. K. Prasad, *et al.* "Multi-Omics Driven Assembly and Annotation of the Sandalwood (*Santalum album*) Genome." *Plant Physiol* 176 (2018): 2772-88.
44. Xu, C. Q., H. Liu, S. S. Zhou, D. X. Zhang, W. Zhao, S. Wang, F. Chen, Y. Q. Sun, S. Nie, K. H. Jia, *et al.* "Genome sequence of *Malania oleifera*, a tree with great value for nervonic acid production." *Gigascience* 8 (2019): giy164.
45. Huang, S., J. Ding, D. Deng, W. Tang, H. Sun, D. Liu, L. Zhang, X. Niu, X. Zhang, M. Meng, *et al.* "Draft genome of the kiwifruit *Actinidia chinensis*." *Nat Commun* 4 (2013): 2640.
46. Pilkington, S. M., R. Crowhurst, E. Hilario, S. Nardozza, L. Fraser, Y. Peng, K. Gunaseelan, R. Simpson, J. Tahir, S. C. Deroles, *et al.* "A manually annotated *Actinidia chinensis* var. chinensis (kiwifruit) genome highlights the challenges associated with draft genomes and gene prediction in plants." *BMC Genomics* 19 (2018): 257.
47. Wu, H., T. Ma, M. Kang, F. Ai, J. Zhang, G. Dong and J. Liu. "A high-quality *Actinidia chinensis* (kiwifruit) genome." *Hortic Res* 6 (2019): 117.
48. Tang, W., X. Sun, J. Yue, X. Tang, C. Jiao, Y. Yang, X. Niu, M. Miao, D. Zhang, S. Huang, *et al.* "Chromosome-scale genome assembly of kiwifruit *Actinidia eriantha* with single-molecule sequencing and chromatin interaction mapping." *Gigascience* 8 (2019): giz027.
49. Zhang, L., P. Xu, Y. Cai, L. Ma, S. Li, S. Li, W. Xie, J. Song, L. Peng, H. Yan, *et al.* "The draft genome assembly of *Rhododendron delavayi* Franch. var. delavayi." *Gigascience* 6 (2017): 1-11.

50. Ma, H., Y. Liu, D. Liu, W. Sun, X. Liu, Y. Wan, X. Zhang, R. Zhang, Q. Yun, J. Wang*, et al.* "Chromosome-level genome assembly and population genetic analysis of a critically endangered rhododendron provide insights into its conservation." *Plant J* (2021): tpj.15399.
51. Shirasawa, K., N. Kobayashi, A. Nakatsuka, H. Ohta and S. Isobe. "Whole-genome sequencing and analysis of two azaleas, *Rhododendron ripense* and *Rhododendron kiyosumense*." *DNA Research* (2021): dsab010.
52. Wang, X., Y. Gao, X. Wu, X. Wen, D. Li, H. Zhou, Z. Li, B. Liu, J. Wei, F. Chen*, et al.* "High-quality evergreen azalea genome reveals tandem duplication-facilitated low-altitude adaptability and floral scent evolution." *Plant Biotechnol J* (2021):
53. Yang, F. S., S. Nie, H. Liu, T. L. Shi, X. C. Tian, S. S. Zhou, Y. T. Bao, K. H. Jia, J. F. Guo, W. Zhao*, et al.* "Chromosome-level genome assembly of a parent species of widely cultivated azaleas." *Nat Commun* 11 (2020): 5269.
54. Soza, V. L., D. Lindsley, A. Waalkes, E. Ramage, R. P. Patwardhan, J. N. Burton, A. Adey, A. Kumar, R. Qiu, J. Shendure*, et al.* "The Rhododendron Genome and Chromosomal Organization Provide Insight into Shared Whole-Genome Duplications across the Heath Family (Ericaceae)." *Genome Biol Evol* 11 (2019): 3353-71.
55. Colle, M., C. P. Leisner, C. M. Wai, S. Ou, K. A. Bird, J. Wang, J. H. Wisecaver, A. E. Yocca, E. I. Alger, H. Tang*, et al.* "Haplotype-phased genome and evolution of phytonutrient pathways of tetraploid blueberry." *Gigascience* 8 (2019): giz012.
56. Polashock, J., E. Zelzion, D. Fajardo, J. Zalapa, L. Georgi, D. Bhattacharya and N. Vorsa. "The American cranberry: first insights into the whole genome of a species adapted to bog habitat." *BMC Plant Biol* 14 (2014): 165.
57. Diaz-Garcia, L., L. F. Garcia-Ortega, M. Gonzalez-Rodriguez, L. Delaye, M. Iorizzo and J. Zalapa. "Chromosome-Level Genome Assembly of the American Cranberry (*Vaccinium macrocarpon* Ait.) and Its Wild Relative *Vaccinium microcarpum*." *Front Plant Sci* 12 (2021): 633310.
58. Wu, C., C. Deng, E. Hilario, N. W. Albert, D. Lafferty, E. R. P. Grierson, B. J. Plunkett, C. Elborough, A. Saei, C. S. Gunther*, et al.* "A chromosome-scale assembly of the bilberry genome identifies a complex locus controlling berry anthocyanin composition." *Mol Ecol Resour* (2021):
59. Ma, D., Z. Guo, Q. Ding, Z. Zhao, Z. Shen, M. Wei, C. Gao, L. Zhang, H. Li, S. Zhang*, et al.* "Chromosome-level assembly of the mangrove plant *Aegiceras corniculatum* genome generated through Illumina, PacBio and Hi-C sequencing technologies." *Mol Ecol Resour* 21 (2021): 1593-607.
60. Akagi, T., K. Shirasawa, H. Nagasaki, H. Hirakawa, R. Tao, L. Comai and I. M. Henry. "The persimmon genome reveals clues to the evolution of a lineage-specific sex determination system in plants." *PLoS Genet* 16 (2020): e1008566.
61. Zhu, Q. G., Y. Xu, Y. Yang, C. F. Guan, Q. Y. Zhang, J. W. Huang, D. Grierson, K. S. Chen, B. C. Gong and X. R. Yin. "The persimmon (*Diospyros oleifera* Cheng) genome provides new insights into the inheritance of astringency and ancestral evolution." *Hortic Res* 6 (2019): 138.
62. Suo, Y., P. Sun, H. Cheng, W. Han, S. Diao, H. Li, Y. Mai, X. Zhao, F. Li and J. Fu. "A high-quality chromosomal genome assembly of *Diospyros oleifera* Cheng." *Gigascience* 9 (2020): giz164.
63. Khayi, S., N. E. Azza, F. Gaboun, S. Pirro, O. Badad, M. G. Claros, D. A. Lightfoot, T. Unver, B. Chaouni, R. Merrouch*, et al.* "First draft genome assembly of the Argane tree (*Argania spinosa*)." *F1000Res* 7 (2018): 1310.
64. Xia, E. H., H. B. Zhang, J. Sheng, K. Li, Q. J. Zhang, C. Kim, Y. Zhang, Y. Liu, T. Zhu, W. Li*, et al.* "The Tea Tree Genome Provides Insights into Tea Flavor and Independent Evolution of Caffeine Biosynthesis." *Mol Plant* 10 (2017): 866-77.
65. Wei, C., H. Yang, S. Wang, J. Zhao, C. Liu, L. Gao, E. Xia, Y. Lu, Y. Tai, G. She*, et al.* "Draft genome sequence of *Camellia sinensis* var. sinensis provides insights into the evolution of the tea genome and tea quality." *P Natl Acad Sci USA* 115 (2018): E4151-E58.

66. Xia, E., F. Li, W. Tong, H. Yang, S. Wang, J. Zhao, C. Liu, L. Gao, Y. Tai, G. She*, et al.* "The tea plant reference genome and improved gene annotation using long-read and paired-end sequencing data." *Sci Data* 6 (2019): 122.
67. Chen, J. D., C. Zheng, J. Q. Ma, C. K. Jiang, S. Ercisli, M. Z. Yao and L. Chen. "The chromosome-scale genome reveals the evolution and diversification after the recent tetraploidization event in tea plant." *Hortic Res* 7 (2020): 63.
68. Wang, X., H. Feng, Y. Chang, C. Ma, L. Wang, X. Hao, A. Li, H. Cheng, L. Wang, P. Cui*, et al.* "Population sequencing enhances understanding of tea plant evolution." *Nat Commun* 11 (2020): 4447.
69. Xia, E., W. Tong, Y. Hou, Y. An, L. Chen, Q. Wu, Y. Liu, J. Yu, F. Li, R. Li*, et al.* "The Reference Genome of Tea Plant and Resequencing of 81 Diverse Accessions Provide Insights into Its Genome Evolution and Adaptation." *Mol Plant* 13 (2020): 1013-26.
70. Zhang, Q. J., W. Li, K. Li, H. Nan, C. Shi, Y. Zhang, Z. Y. Dai, Y. L. Lin, X. L. Yang, Y. Tong*, et al.* "The Chromosome-Level Reference Genome of Tea Tree Unveils Recent Bursts of Non-autonomous LTR Retrotransposons in Driving Genome Size Evolution." *Mol Plant* 13 (2020): 935-38.
71. Zhang, W., Y. Zhang, H. Qiu, Y. Guo, H. Wan, X. Zhang, F. Scossa, S. Alseekh, Q. Zhang, P. Wang*, et al.* "Genome assembly of wild tea tree DASZ reveals pedigree and selection history of tea varieties." *Nat Commun* 11 (2020): 3719.
72. Zhang, X., S. Chen, L. Shi, D. Gong, S. Zhang, Q. Zhao, D. Zhan, L. Vasseur, Y. Wang, J. Yu*, et al.* "Haplotype-resolved genome assembly provides insights into evolutionary history of the tea plant *Camellia sinensis*." *Nat Genet* 53 (2021): 1250-59.
73. Zhao, D., J. P. Hamilton, G. M. Pham, E. Crisovan, K. Wiegert-Rininger, B. Vaillancourt, D. DellaPenna and C. R. Buell. "De novo genome assembly of *Camptotheca acuminata*, a natural source of the anti-cancer compound camptothecin." *Gigascience* 6 (2017): 1-7.
74. Kang, M., R. Fu, P. Zhang, S. Lou, X. Yang, Y. Chen, T. Ma, Y. Zhang, Z. Xi and J. Liu. "A chromosome-level *Camptotheca acuminata* genome assembly provides insights into the evolutionary origin of camptothecin biosynthesis." *Nat Commun* 12 (2021): 3531.
75. Chen, Y., T. Ma, L. Zhang, M. Kang, Z. Zhang, Z. Zheng, P. Sun, N. Shrestha, J. Liu and Y. Yang. "Genomic analyses of a "living fossil": The endangered dove-tree." *Mol Ecol Resour* 20 (2020): 1755-0998.13138.
76. Yang, X., M. Kang, Y. Yang, H. Xiong, M. Wang, Z. Zhang, Z. Wang, H. Wu, T. Ma, J. Liu*, et al.* "A chromosome-level genome assembly of the Chinese tupelo *Nyssa sinensis*." *Sci. Data* 6 (2019): s41597-019-0296-y.
77. Mu, W., J. Wei, T. Yang, Y. Fan, L. Cheng, J. Yang, R. Mu, J. Liu, J. Zhao, W. Sun*, et al.* "The draft genome assembly of the critically endangered *Nyssa yunnanensis*, a plant species with extremely small populations endemic to Yunnan Province, China." *Gigabyte* 2020 (2020): 1-12.
78. Cao, Y., G. Sun, X. Zhai, P. Xu, L. Ma, M. Deng, Z. Zhao, H. Yang, Y. Dong, Z. Shang*, et al.* "Genomic insights into the fast growth of paulownias and the formation of Paulownia witches' broom." *Mol Plant* (2021):
79. Natarajan, P., A. K. Murugesan, G. Govindan, A. Gopalakrishnan, R. Kumar, P. Duraisamy, R. Balaji, Tanuja, P. S. Shyamli, A. K. Parida*, et al.* "A reference-grade genome identifies salt-tolerance genes from the salt-secreting mangrove species *Avicennia marina*." *Commun Biol* 4 (2021): 851.
80. Friis, G., J. Vizueta, E. G. Smith, D. R. Nelson, B. Khraiwesh, E. Qudeimat, K. Salehi-Ashtiani, A. Ortega, A. Marshell, C. M. Duarte*, et al.* "A high-quality genome assembly and annotation of the gray mangrove, *Avicennia marina*." *G3 (Bethesda)* 11 (2021): jkaa025.
81. Li, L. F., S. A. Cushman, Y. X. He and Y. Li. "Genome sequencing and population genomics modeling provide insights into the local adaptation of weeping forsythia." *Hortic Res* 7 (2020): 130.
82. Sollars, E. S., A. L. Harper, L. J. Kelly, C. M. Sambles, R. H. Ramirez-Gonzalez, D. Swarbreck, G. Kaithakottil, E. D. Cooper, C. Uauy, L. Havlickova*, et al.* "Genome sequence and genetic diversity of European ash trees." *Nature* 541 (2017): 212-16.

83. Cruz, F., I. Julca, J. Gómez-Garrido, D. Loska, M. Marcet-Houben, E. Cano, B. Galán, L. Frias, P. Ribeca, S. Derdak*, et al.* "Genome sequence of the olive tree, *Olea europaea*." *GigaScience* 5 (2016): s13742-016-0134-5.
84. Unver, T., Z. Wu, L. Sterck, M. Turktas, R. Lohaus, Z. Li, M. Yang, L. He, T. Deng, F. J. Escalante*, et al.* "Genome of wild olive and the evolution of oil biosynthesis." *P Natl Acad Sci USA* 114 (2017): E9413-E22.
85. Rao, G., J. Zhang, X. Liu, C. Lin, H. Xin, L. Xue and C. Wang. "De novo assembly of a new *Olea europaea* genome accession using nanopore sequencing." *Hortic Res* 8 (2021): 64.
86. Yang, X., Y. Yue, H. Li, W. Ding, G. Chen, T. Shi, J. Chen, M. S. Park, F. Chen and L. Wang. "The chromosome-level quality genome provides insights into the evolution of the biosynthesis genes for aroma compounds of *Osmanthus fragrans*." *Hortic Res* 6 (2019): 62.
87. Chen, H., X. Zeng, J. Yang, X. Cai, Y. Shi, R. Zheng, Z. Wang, J. Liu, X. Yi, S. Xiao*, et al.* "Whole-genome resequencing of *Osmanthus fragrans* provides insights into flower color evolution." *Hortic Res* 8 (2021): 98.
88. Burley, J. T., J. R. Kellner, S. P. Hubbell and B. C. Faircloth. "Genome assemblies for two Neotropical trees: *Jacaranda copaia* and *Handroanthus guayacan*." *G3 (Bethesda)* 11 (2021): jkab010.
89. Silva-Junior, O. B., D. Grattapaglia, E. Novaes and R. G. Collevatti. "Genome assembly of the Pink Ipê (*Handroanthus impetiginosus*, Bignoniaceae), a highly valued, ecologically keystone Neotropical timber forest tree." *GigaScience* 7 (2018): gix125.
90. Wang, M., L. Zhang and Z. Wang. "Chromosomal-Level Reference Genome of the Neotropical Tree *Jacaranda mimosifolia* D. Don." *Genome Biol Evol* 13 (2021):
91. Yasodha, R., R. Vasudeva, S. Balakrishnan, A. R. Sakthi, N. Abel, N. Binai, B. Rajashekar, V. K. W. Bachpai, C. Pillai and S. A. Dev. "Draft genome of a high value tropical timber tree, Teak (*Tectona grandis* L. f): insights into SSR diversity, phylogeny and conservation." *DNA Research* 25 (2018): 409-19.
92. Zhao, D., J. P. Hamilton, W. W. Bhat, S. R. Johnson, G. T. Godden, T. J. Kinser, B. Boachon, N. Dudareva, D. E. Soltis, P. S. Soltis*, et al.* "A chromosomal-scale genome assembly of *Tectona grandis* reveals the importance of tandem gene duplication and enables discovery of genes in natural product biosynthetic pathways." *GigaScience* 8 (2019): giz005.
93. Cao, Y.-L., Y.-L. Li, Y.-F. Fan, Z. Li, K. Yoshida, J.-Y. Wang, X.-K. Ma, N. Wang, N. Mitsuda, T. Kotake*, et al.* "Wolfberry genomes and the evolution of Lycium (Solanaceae)." *Commun Biol* 4 (2021):
94. Tran, H. T. M., T. Ramaraj, A. Furtado, L. S. Lee and R. J. Henry. "Use of a draft genome of coffee (*Coffea arabica*) to identify SNP s associated with caffeine content." *Plant Biotechnol J* 16 (2018): 1756-66.
95. Denoeud, F., L. Carretero-Paulet, A. Dereeper, G. Droc, R. Guyot, M. Pietrella, C. Zheng, A. Alberti, F. Anthony, G. Aprea*, et al.* "The coffee genome provides insight into the convergent evolution of caffeine biosynthesis." *Science* 345 (2014): 1181-4.
96. Raharimalala, N., S. Rombauts, A. McCarthy, A. Garavito, S. Orozco-Arias, L. Bellanger, A. Y. Morales-Correa, S. Froger, S. Michaux, V. Berry*, et al.* "The absence of the caffeine synthase gene is involved in the naturally decaffeinated status of *Coffea humblotiana*, a wild species from *Comoro archipelago*." *Sci Rep-Uk* 11 (2021): s41598-021-87419-0.
97. Brose, J., K. H. Lau, T. T. T. Dang, J. P. Hamilton, L. D. V. Martins, B. Hamberger, B. Hamberger, J. Jiang, S. E. O'Connor and C. R. Buell. "The *Mitragyna speciosa* (Kratom) Genome: a resource for data-mining potent pharmaceuticals that impact human health." *G3 (Bethesda)* 11 (2021): jkab058.
98. Li, Y., H. Wei, J. Yang, K. Du, J. Li, Y. Zhang, T. Qiu, Z. Liu, Y. Ren, L. Song*, et al.* "High-quality de novo assembly of the *Eucommia ulmoides* haploid genome provides new insights into evolution and rubber biosynthesis." *Hortic Res* 7 (2020): 183.
99. Wuyun, T. N., L. Wang, H. Liu, X. Wang, L. Zhang, J. L. Bennetzen, T. Li, L. Yang, P. Liu, L. Du*, et al.* "The Hardy Rubber Tree Genome Provides Insights into the Evolution of Polyisoprene Biosynthesis." *Mol Plant* 11 (2018): 429-42.

100. Wang, Y., H. Xin, P. Fan, J. Zhang, Y. Liu, Y. Dong, Z. Wang, Y. Yang, Q. Zhang, R. Ming, *et al.* "The genome of Shanputao (*Vitis amurensis*) provides a new insight into cold tolerance of grapevine." *Plant J* 105 (2021): 1495-506.
101. Patel, S., M. Robben, A. Fennell, J. P. Londo, D. Alahakoon, R. Villegas-Diaz and P. Swaminathan. "Draft genome of the Native American cold hardy grapevine *Vitis riparia* Michx. 'Manitoba 37'." *Hortic Res* 7 (2020): 92.
102. Girollet, N., B. Rubio, C. Lopez-Roques, S. Valiere, N. Ollat and P. F. Bert. "De novo phased assembly of the *Vitis riparia* grape genome." *Sci Data* 6 (2019): 127.
103. Jaillon, O., J. M. Aury, B. Noel, A. Policriti, C. Clepet, A. Casagrande, N. Choisne, S. Aubourg, N. Vitulo, C. Jubin, *et al.* "The grapevine genome sequence suggests ancestral hexaploidization in major angiosperm phyla." *Nature* 449 (2007): 463-7.
104. Ramos, M. J. N., J. L. Coito, D. Faisca-Silva, J. Cunha, M. M. R. Costa, S. Amancio and M. Rocheta. "Portuguese wild grapevine genome re-sequencing (*Vitis vinifera* sylvestris)." *Sci Rep* 10 (2020): 18993.
105. Badouin, H., A. Velt, F. Gindraud, T. Flutre, V. Dumas, S. Vautrin, W. Marande, J. Corbi, E. Sallet, J. Ganofsky, *et al.* "The wild grape genome sequence provides insights into the transition from dioecy to hermaphroditism during grape domestication." *Genome Biol* 21 (2020): s13059-020-02131-y.
106. Liang, Z., S. Duan, J. Sheng, S. Zhu, X. Ni, J. Shao, C. Liu, P. Nick, F. Du, P. Fan, *et al.* "Whole-genome resequencing of 472 Vitis accessions for grapevine diversity and demographic history analyses." *Nat Commun* 10 (2019): s41467-019-09135-8.
107. Cochetel, N., A. Minio, M. Massonnet, A. M. Vondras, R. Figueroa-Balderas and D. Cantu. "Diploid chromosome-scale assembly of the *Muscadinia rotundifolia* genome supports chromosome fusion and disease resistance gene expansion during Vitis and Muscadinia divergence." *G3 (Bethesda)* 11 (2021): jkab033.
108. Hong, Z., J. Li, X. Liu, J. Lian, N. Zhang, Z. Yang, Y. Niu, Z. Cui and D. Xu. "The chromosome-level draft genome of *Dalbergia odorifera*." *Gigascience* 9 (2020): giaa084.
109. Chang, Y., H. Liu, M. Liu, X. Liao, S. K. Sahu, Y. Fu, B. Song, S. Cheng, R. Kariba, S. Muthemba, *et al.* "The draft genomes of five agriculturally important African orphan crops." *GigaScience* 8 (2019): giy152.
110. Soyturk, A., F. Sen, A. T. Uncu, I. Celik and A. O. Uncu. "De novo assembly and characterization of the first draft genome of quince (*Cydonia oblonga* Mill.)." *Sci Rep-Uk* 11 (2021):
111. Jiang, S., H. An, F. Xu and X. Zhang. "Chromosome-level genome assembly and annotation of the loquat (*Eriobotrya japonica*) genome." *GigaScience* 9 (2020): giaa015.
112. Su, W., Y. Jing, S. Lin, Z. Yue, X. Yang, J. Xu, J. Wu, Z. Zhang, R. Xia, J. Zhu, *et al.* "Polyploidy underlies co-option and diversification of biosynthetic triterpene pathways in the apple tribe." *P Natl Acad Sci USA* 118 (2021): e2101767118.
113. Chen, X., S. Li, D. Zhang, M. Han, X. Jin, C. Zhao, S. Wang, L. Xing, J. Ma, J. Ji, *et al.* "Sequencing of a Wild Apple (*Malus baccata*) Genome Unravels the Differences Between Cultivated and Wild Apple Species Regarding Disease Resistance and Cold Tolerance." *G3 (Bethesda)* 9 (2019): 2051-60.
114. Velasco, R., A. Zharkikh, J. Affourtit, A. Dhingra, A. Cestaro, A. Kalyanaraman, P. Fontana, S. K. Bhatnagar, M. Troggio, D. Pruss, *et al.* "The genome of the domesticated apple (*Malus × domestica* Borkh.)." *Nat Genet* 42 (2010): 833-39.
115. Duan, N., Y. Bai, H. Sun, N. Wang, Y. Ma, M. Li, X. Wang, C. Jiao, N. Legall, L. Mao, *et al.* "Genome re-sequencing reveals the history of apple and supports a two-stage model for fruit enlargement." *Nat Commun* 8 (2017): s41467-017-00336-7.
116. Daccord, N., J.-M. Celton, G. Linsmith, C. Becker, N. Choisne, E. Schijlen, H. Van De Geest, L. Bianco, D. Micheletti, R. Velasco, *et al.* "High-quality de novo assembly of the apple genome and methylome dynamics of early fruit development." *Nat Genet* 49 (2017): 1099-106.

117. Zhang, L., J. Hu, X. Han, J. Li, Y. Gao, C. M. Richards, C. Zhang, Y. Tian, G. Liu, H. Gul*, et al.* "A high-quality apple genome assembly reveals the association of a retrotransposon and red fruit colour." *Nat Commun* 10 (2019): s41467-019-09518-x.
118. Sun, X., C. Jiao, H. Schwaninger, C. T. Chao, Y. Ma, N. Duan, A. Khan, S. Ban, K. Xu, L. Cheng*, et al.* "Phased diploid genome assemblies and pan-genomes provide insights into the genetic history of apple domestication." *Nat Genet* 52 (2020): 1423-32.
119. Groppi, A., S. Liu, A. Cornille, S. Decroocq, Q. T. Bui, D. Tricon, C. Cruaud, S. Arribat, C. Belser, W. Marande*, et al.* "Population genomics of apricots unravels domestication history and adaptive events." *Nat Commun* 12 (2021): s41467-021-24283-6.
120. Pinosio, S., F. Marroni, A. Zuccolo, N. Vitulo, S. Mariette, G. Sonnante, F. A. Aravanopoulos, I. Ganopoulos, M. Palasciano, M. Vidotto*, et al.* "A draft genome of sweet cherry (*Prunus avium*L.) reveals genome-wide and local effects of domestication." *Plant J* 103 (2020): 1420-32.
121. Shirasawa, K., K. Isuzugawa, M. Ikenaga, Y. Saito, T. Yamamoto, H. Hirakawa and S. Isobe. "The genome sequence of sweet cherry (*Prunus avium*) for use in genomics-assisted breeding." *DNA Research* 24 (2017): 499-508.
122. Wang, J., W. Liu, D. Zhu, P. Hong, S. Zhang, S. Xiao, Y. Tan, X. Chen, L. Xu, X. Zong*, et al.* "Chromosome-scale genome assembly of sweet cherry (*Prunus avium* L.) cv. Tieton obtained using long-read and Hi-C sequencing." *Hortic Res* 7 (2020): s41438-020-00343-8.
123. Wang, J., W. Liu, D. Zhu, X. Zhou, P. Hong, H. Zhao, Y. Tan, X. Chen, X. Zong, L. Xu*, et al.* "A de novo assembly of the sweet cherry (*Prunus avium* cv. Tieton) genome using linked-read sequencing technology." *Peerj* 8 (2020): e9114.
124. Callahan, A. M., T. N. Zhebentyayeva, J. L. Humann, C. A. Saski, K. D. Galimba, L. L. Georgi, R. Scorza, D. Main and C. D. Dardick. "Defining the 'HoneySweet' insertion event utilizing NextGen sequencing and a de novo genome assembly of plum (*Prunus domestica*)." *Hortic Res* 8 (2021): s41438-020-00438-2.
125. Alioto, T., K. G. Alexiou, A. Bardil, F. Barteri, R. Castanera, F. Cruz, A. Dhingra, H. Duval, Á. Fernández I Martí, L. Frias*, et al.* "Transposons played a major role in the diversification between the closely related almond and peach genomes: results from the almond genome sequence." *Plant J* 101 (2020): 455-72.
126. Wang, P., S. Yi, X. Mu, J. Zhang and J. Du. "Chromosome-Level Genome Assembly of *Cerasus humilis* Using PacBio and Hi-C Technologies." *Front Genet* 11 (2020): 956.
127. Zhang, Q., W. Chen, L. Sun, F. Zhao, B. Huang, W. Yang, Y. Tao, J. Wang, Z. Yuan, G. Fan*, et al.* "The genome of *Prunus mume*." *Nat Commun* 3 (2012): 1318.
128. Verde, I., A. G. Abbott, S. Scalabrin, S. Jung, S. Shu, F. Marroni, T. Zhebentyayeva, M. T. Dettori, J. Grimwood, F. Cattonaro*, et al.* "The high-quality draft genome of peach (*Prunus persica*) identifies unique patterns of genetic diversity, domestication and genome evolution." *Nat Genet* 45 (2013): 487-94.
129. Guan, J., Y. Xu, Y. Yu, J. Fu, F. Ren, J. Guo, J. Zhao, Q. Jiang, J. Wei and H. Xie. "Genome structure variation analyses of peach reveal population dynamics and a 1.67 Mb causal inversion for fruit shape." *Genome Biol* 22 (2021): s13059-020-02239-1.
130. Zhang, A., H. Zhou, X. Jiang, Y. Han and X. Zhang. "The Draft Genome of a Flat Peach (*Prunus persica* L. cv. '124 Pan') Provides Insights into Its Good Fruit Flavor Traits." *Plants* 10 (2021): 538.
131. Huang, Z., F. Shen, Y. Chen, K. Cao and L. Wang. "Chromosome-scale genome assembly and population genomics provide insights into the adaptation, domestication, and flavonoid metabolism of Chinese plum." *Plant J* (2021):
132. Liu, C., C. Feng, W. Peng, J. Hao, J. Wang, J. Pan and Y. He. "Chromosome-level draft genome of a diploid plum (*Prunus salicina*)." *GigaScience* 9 (2020): giaa130.
133. Yi, X.-G., X.-Q. Yu, J. Chen, M. Zhang, S.-W. Liu, H. Zhu, M. Li, Y.-F. Duan, L. Chen, L. Wu*, et al.* "The genome of Chinese flowering cherry (*Cerasus serrulata*) provides new insights into Cerasus species." *Hortic Res* 7 (2020): s41438-020-00382-1.
134. Baek, S., K. Choi, G.-B. Kim, H.-J. Yu, A. Cho, H. Jang, C. Kim, H.-J. Kim, K. S. Chang, J.-H. Kim*, et al.* "Draft genome sequence of wild *Prunus yedoensis* reveals massive inter-

specific hybridization between sympatric flowering cherries." *Genome Biol* 19 (2018): s13059-018-1497-y.

135. Sahu, S., M. Liu, A. Yssel, R. Kariba, S. Muthemba, S. Jiang, B. Song, P. Hendre, A. Muchugi, R. Jamnadass*, et al.* "Draft Genomes of Two Artocarpus Plants, Jackfruit (*A. heterophyllus*) and Breadfruit (*A. altilis*)." *Genes* 11 (2019): 27.

136. Gardner, E. M., M. G. Johnson, D. Ragone, N. J. Wickett and N. J. C. Zerega. "Low-coverage, whole-genome sequencing of *Artocarpus camansi* (Moraceae) for phylogenetic marker development and gene discovery." *Applications in Plant Sciences* 4 (2016): 1600017.

137. Mori, K., K. Shirasawa, H. Nogata, C. Hirata, K. Tashiro, T. Habu, S. Kim, S. Himeno, S. Kuhara and H. Ikegami. "Identification of RAN1 orthologue associated with sex determination through whole genome sequencing analysis in fig (*Ficus carica* L.)." *Sci Rep-Uk* 7 (2017): 41124.

138. Shirasawa, K., H. Yakushiji, R. Nishimura, T. Morita, S. Jikumaru, H. Ikegami, A. Toyoda, H. Hirakawa and S. Isobe. "The *Ficus erecta* genome aids *Ceratocystis canker* resistance breeding in common fig (*F. carica*)." *Plant J* 102 (2020): 1313-22.

139. Zhang, X., G. Wang, S. Zhang, S. Chen, Y. Wang, P. Wen, X. Ma, Y. Shi, R. Qi, Y. Yang*, et al.* "Genomes of the Banyan Tree and Pollinator Wasp Provide Insights into Fig-Wasp Coevolution." *Cell* 183 (2020): 875-89 e17.

140. Ma, J., D. Wan, B. Duan, X. Bai, Q. Bai, N. Chen and T. Ma. "Genome sequence and genetic transformation of a widely distributed and cultivated poplar." *Plant Biotechnol J* 17 (2019): 451-60.

141. Mader, M., M.-C. Le Paslier, R. Bounon, A. Bérard, P. F. Rampant, M. Fladung, J.-C. Leplé and B. Kersten. "Whole-genome draft assembly of *Populus tremula* x *P. alba* clone INRA 717-1B4." *Silvae Genetica* 65 (2016): 74-79.

142. Ma, T., J. Wang, G. Zhou, Z. Yue, Q. Hu, Y. Chen, B. Liu, Q. Qiu, Z. Wang, J. Zhang*, et al.* "Genomic insights into salt adaptation in a desert poplar." *Nat Commun* 4 (2013): 2797.

143. Huang, X., S. Chen, X. Peng, E.-K. Bae, X. Dai, G. Liu, G. Qu, J.-H. Ko, H. Lee, S. Chen*, et al.* "An improved draft genome sequence of hybrid *Populus alba* × *Populus glandulosa*." *Journal of Forestry Research* 32 (2021): 1663-72.

144. Qiu, D., S. Bai, J. Ma, L. Zhang, F. Shao, K. Zhang, Y. Yang, T. Sun, J. Huang, Y. Zhou*, et al.* "The genome of *Populus alba* x *Populus tremula* var. glandulosa clone 84K." *DNA Res* 26 (2019): 423-31.

145. Chen, Z., F. Ai, J. Zhang, X. Ma, W. Yang, W. Wang, Y. Su, M. Wang, Y. Yang, K. Mao*, et al.* "Survival in the Tropics despite isolation, inbreeding and asexual reproduction: insights from the genome of the world's southernmost poplar (*Populus ilicifolia*)." *Plant J* 103 (2020): 430-42.

146. Yang, W., K. Wang, J. Zhang, J. Ma, J. Liu and T. Ma. "The draft genome sequence of a desert tree *Populus pruinosa*." *Gigascience* 6 (2017): 1-7.

147. Lin, Y. C., J. Wang, N. Delhomme, B. Schiffthaler, G. Sundstrom, A. Zuccolo, B. Nystedt, T. R. Hvidsten, A. de la Torre, R. M. Cossu*, et al.* "Functional and evolutionary genomic inferences in Populus through genome and population sequencing of American and European aspen." *P Natl Acad Sci USA* 115 (2018): E10970-E78.

148. Tuskan, G. A., S. Difazio, S. Jansson, J. Bohlmann, I. Grigoriev, U. Hellsten, N. Putnam, S. Ralph, S. Rombauts, A. Salamov*, et al.* "The genome of black cottonwood, *Populus trichocarpa* (Torr. & Gray)." *Science* 313 (2006): 1596-604.

149. Chen, J. H., Y. Huang, B. Brachi, Q. Z. Yun, W. Zhang, W. Lu, H. N. Li, W. Q. Li, X. D. Sun, G. Y. Wang*, et al.* "Genome-wide analysis of Cushion willow provides insights into alpine plant divergence in a biodiversity hotspot." *Nat Commun* 10 (2019): 5230.

150. He, L., K. H. Jia, R. G. Zhang, Y. Wang, T. L. Shi, Z. C. Li, S. W. Zeng, X. J. Cai, N. D. Wagner, E. Hörandl*, et al.* "Chromosome-scale assembly of the genome of *Salix dunnii* reveals a male-heterogametic sex determination system on chromosome 7." *Mol. Ecol. Resour.* 21 (2021): 1966-82.

151. Zhang, J., H. Yuan, Y. Li, Y. Chen, G. Liu, M. Ye, C. Yu, B. Lian, F. Zhong, Y. Jiang, *et al.* "Genome sequencing and phylogenetic analysis of allotetraploid *Salix matsudana* Koidz." *Hortic Res* 7 (2020): 201.
152. Dai, X., Q. Hu, Q. Cai, K. Feng, N. Ye, G. A. Tuskan, R. Milne, Y. Chen, Z. Wan, Z. Wang, *et al.* "The willow genome and divergent evolution from poplar after the common genome duplication." *Cell Res* 24 (2014): 1274-7.
153. Wei, S., Y. Yang and T. Yin. "The chromosome-scale assembly of the willow genome provides insight into Salicaceae genome evolution." *Hortic Res* 7 (2020): 45.
154. Almeida, P., E. Proux-Wera, A. Churcher, L. Soler, J. Dainat, P. Pucholt, J. Nordlund, T. Martin, A. C. Ronnberg-Wastljung, B. Nystedt, *et al.* "Genome assembly of the basket willow, *Salix viminalis*, reveals earliest stages of sex chromosome expansion." *BMC Biol* 18 (2020): 78.
155. Rahman, A. Y. A., A. O. Usharraj, B. B. Misra, G. P. Thottathil, K. Jayasekaran, Y. Feng, S. Hou, S. Y. Ong, F. L. Ng, L. S. Lee, *et al.* "Draft genome sequence of the rubber tree *Hevea brasiliensis*." *BMC Genomics* 14 (2013): 75.
156. Tang, C., M. Yang, Y. Fang, Y. Luo, S. Gao, X. Xiao, Z. An, B. Zhou, B. Zhang, X. Tan, *et al.* "The rubber tree genome reveals new insights into rubber production and species adaptation." *Nat Plants* 2 (2016): 16073.
157. Lau, N.-S., Y. Makita, M. Kawashima, T. D. Taylor, S. Kondo, A. S. Othman, A. C. Shu-Chien and M. Matsui. "The rubber tree genome shows expansion of gene family associated with rubber biosynthesis." *Sci Rep-Uk* 6 (2016): 28594.
158. Pootakham, W., C. Sonthirod, C. Naktang, P. Ruang-Areerate, T. Yoocha, D. Sangsrakru, K. Theerawattanasuk, R. Rattanawong, N. Lekawipat and S. Tangphatsornruang. "De novo hybrid assembly of the rubber tree genome reveals evidence of paleotetraploidy in Hevea species." *Sci Rep-Uk* 7 (2017): 41457.
159. Liu, J., C. Shi, C. C. Shi, W. Li, Q. J. Zhang, Y. Zhang, K. Li, H. F. Lu, C. Shi, S. T. Zhu, *et al.* "The Chromosome-Based Rubber Tree Genome Provides New Insights into Spurge Genome Evolution and Rubber Biosynthesis." *Mol Plant* 13 (2020): 336-50.
160. Ha, J., S. Shim, T. Lee, Y. J. Kang, W. J. Hwang, H. Jeong, K. Laosatit, J. Lee, S. K. Kim, D. Satyawan, *et al.* "Genome sequence of *Jatropha curcas* L., a non-edible biodiesel plant, provides a resource to improve seed-related traits." *Plant Biotechnol J* 17 (2019): 517-30.
161. Wu, P., C. Zhou, S. Cheng, Z. Wu, W. Lu, J. Han, Y. Chen, Y. Chen, P. Ni, Y. Wang, *et al.* "Integrated genome sequence and linkage map of physic nut (*Jatropha curcas* L.), a biodiesel plant." *Plant J* 81 (2015): 810-21.
162. Sato, S., H. Hirakawa, S. Isobe, E. Fukai, A. Watanabe, M. Kato, K. Kawashima, C. Minami, A. Muraki, N. Nakazaki, *et al.* "Sequence Analysis of the Genome of an Oil-Bearing Tree, *Jatropha curcas* L." *DNA Research* 18 (2011): 65-76.
163. Cui, P., Q. Lin, D. Fang, L. Zhang, R. Li, J. Cheng, F. Gao, J. Shockey, S. Hu and S. Lï¿½. "Tung Tree (*Vernicia fordii,* Hemsl.) Genome and Transcriptome Sequencing Reveals Co-Ordinate Up-Regulation of Fatty Acid β-Oxidation and Triacylglycerol Biosynthesis Pathways During Eleostearic Acid Accumulation in Seeds." *Plant Cell Physiol* 59 (2018): 1990-2003.
164. Zhang, L., M. Liu, H. Long, W. Dong, A. Pasha, E. Esteban, W. Li, X. Yang, Z. Li, A. Song, *et al.* "Tung Tree (*Vernicia fordii*) Genome Provides A Resource for Understanding Genome Evolution and Improved Oil Production." *Genomics, proteomics & bioinformatics* 17 (2019): 558-75.
165. Huang, Y., L. Xiao, Z. Zhang, R. Zhang, Z. Wang, C. Huang, R. Huang, Y. Luan, T. Fan, J. Wang, *et al.* "The genomes of pecan and Chinese hickory provide insights into Carya evolution and nut nutrition." *GigaScience* 8 (2019): giz036.
166. Lovell, J. T., N. B. Bentley, G. Bhattarai, J. W. Jenkins, A. Sreedasyam, Y. Alarcon, C. Bock, L. B. Boston, J. Carlson, K. Cervantes, *et al.* "Four chromosome scale genomes and a pan-genome annotation to accelerate pecan tree breeding." *Nat Commun* 12 (2021):

167. Stevens, K. A., K. Woeste, S. Chakraborty, M. W. Crepeau, C. A. Leslie, P. J. Martínez-García, D. Puiu, J. Romero-Severson, M. Coggeshall, A. M. Dandekar, *et al.* "Genomic Variation Among and Within Six Juglans Species." *G3 (Bethesda)* 8 (2018): 2153-65.
168. Yan, F., R. M. Xi, R. X. She, P. P. Chen, Y. J. Yan, G. Yang, M. Dang, M. Yue, D. Pei, K. Woeste, *et al.* "Improved de novo chromosome-level genome assembly of the vulnerable walnut tree *Juglans mandshurica* reveals gene family evolution and possible genome basis of resistance to lesion nematode." *Mol. Ecol. Resour.* 21 (2021): 2063-76.
169. Zhu, T., L. Wang, F. M. You, J. C. Rodriguez, K. R. Deal, L. Chen, J. Li, S. Chakraborty, B. Balan, C.-Z. Jiang, *et al.* "Sequencing a *Juglans regia* × *J. microcarpa* hybrid yields high-quality genome assemblies of parental species." *Hortic Res* 6 (2019): s41438-019-0139-1.
170. Marrano, A., M. Britton, P. A. Zaini, A. V. Zimin, R. E. Workman, D. Puiu, L. Bianco, E. A. D. Pierro, B. J. Allen, S. Chakraborty, *et al.* "High-quality chromosome-scale assembly of the walnut (*Juglans regia* L.) reference genome." *GigaScience* 9 (2020): giaa050.
171. Ning, D.-L., T. Wu, L.-J. Xiao, T. Ma, W.-L. Fang, R.-Q. Dong and F.-L. Cao. "Chromosomal-level assembly of *Juglans sigillata* genome using Nanopore, BioNano, and Hi-C analysis." *GigaScience* 9 (2020): giaa006.
172. Griesmann, M., Y. Chang, X. Liu, Y. Song, G. Haberer, M. B. Crook, B. Billault-Penneteau, D. Lauressergues, J. Keller, L. Imanishi, *et al.* "Phylogenomics reveals multiple losses of nitrogen-fixing root nodule symbiosis." *Science* 361 (2018): science.aat1743.
173. Wang, N., M. Thomson, W. J. A. Bodles, R. M. M. Crawford, H. V. Hunt, A. W. Featherstone, J. Pellicer and R. J. A. Buggs. "Genome sequence of dwarf birch (*Betula nana*) and cross-species RAD markers." *Molecular Ecology* 22 (2013): 3098-111.
174. Salojarvi, J., O. P. Smolander, K. Nieminen, S. Rajaraman, O. Safronov, P. Safdari, A. Lamminmaki, J. Immanen, T. Lan, J. Tanskanen, *et al.* "Genome sequencing and population genomic analyses provide insights into the adaptive landscape of silver birch." *Nat Genet* 49 (2017): 904-12.
175. Chen, S., Y. Wang, L. Yu, T. Zheng, S. Wang, Z. Yue, J. Jiang, S. Kumari, C. Zheng, H. Tang, *et al.* "Genome sequence and evolution of *Betula platyphylla*." *Hortic Res* 8 (2021): s41438-021-00481-7.
176. Yang, X., Z. Wang, L. Zhang, G. Hao, J. Liu and Y. Yang. "A chromosome-level reference genome of the hornbeam, *Carpinus fangiana*." *Sci. Data* 7 (2020): s41597-020-0370-5.
177. Lucas, S. J., K. Kahraman, B. Avşar, R. J. A. Buggs and I. Bilge. "A chromosome-scale genome assembly of European hazel (*Corylus avellana* L.) reveals targets for crop improvement." *Plant J* 105 (2021): 1413-30.
178. Pavese, V., E. Cavalet-Giorsa, L. Barchi, A. Acquadro, D. Torello Marinoni, E. Portis, S. J. Lucas and R. Botta. "Whole-genome assembly of Corylus avellana cv “Tonda Gentile delle Langhe” using linked-reads (10X Genomics)." *G3 Genes|Genomes|Genetics* 11 (2021):
179. Zhao, T., W. Ma, Z. Yang, L. Liang, X. Chen, G. Wang, Q. Ma and L. Wang. "A chromosome-level reference genome of the hazelnut, *Corylus heterophylla* Fisch." *GigaScience* 10 (2021): giab027.
180. Li, Y., P. Sun, Z. Lu, J. Chen, Z. Wang, X. Du, Z. Zheng, Y. Wu, H. Hu, J. Yang, *et al.* "The Corylus mandshurica genome provides insights into the evolution of Betulaceae genomes and hazelnut breeding." *Hortic Res* 8 (2021):
181. Yang, Y., T. Ma, Z. Wang, Z. Lu, Y. Li, C. Fu, X. Chen, M. Zhao, M. S. Olson and J. Liu. "Genomic effects of population collapse in a critically endangered ironwood tree *Ostrya rehderiana*." *Nat Commun* 9 (2018): s41467-018-07913-4.
182. Ye, G., H. Zhang, B. Chen, S. Nie, H. Liu, W. Gao, H. Wang, Y. Gao and L. Gu. "De novo genome assembly of the stress tolerant forest species Casuarina equisetifolia provides insight into secondary growth." *Plant J* 97 (2019): 779-94.
183. Jia, H.-M., H.-J. Jia, Q.-L. Cai, Y. Wang, H.-B. Zhao, W.-F. Yang, G.-Y. Wang, Y.-H. Li, D.-L. Zhan, Y.-T. Shen, *et al.* "The red bayberry genome and genetic basis of sex determination." *Plant Biotechnol J* 17 (2019): 397-409.

184. Ren, H., H. Yu, S. Zhang, S. Liang, X. Zheng, S. Zhang, P. Yao, H. Zheng and X. Qi. "Genome sequencing provides insights into the evolution and antioxidant activity of Chinese bayberry." *BMC Genomics* 20 (2019):

185. Shirasawa, K., S. Nishio, S. Terakami, R. Botta, D. T. Marinoni and S. Isobe. "Chromosome-level genome assembly of Japanese chestnut (*Castanea crenata* Sieb. et Zucc.) reveals conserved chromosomal segments in woody rosids." *DNA Research* 28 (2021):

186. Xing, Y., Y. Liu, Q. Zhang, X. Nie, Y. Sun, Z. Zhang, H. Li, K. Fang, G. Wang, H. Huang*, et al.* "Hybrid de novo genome assembly of Chinese chestnut (*Castanea mollissima*)." *GigaScience* 8 (2019): giz112.

187. Mishra, B., D. K. Gupta, M. Pfenninger, T. Hickler, E. Langer, B. Nam, J. Paule, R. Sharma, B. Ulaszewski, J. Warmbier*, et al.* "A reference genome of the European beech (*Fagus sylvatica* L.)." *GigaScience* 7 (2018): giy063.

188. Sork, V. L., S. T. Fitz-Gibbon, D. Puiu, M. Crepeau, P. F. Gugger, R. Sherman, K. Stevens, C. H. Langley, M. Pellegrini and S. L. Salzberg. "First Draft Assembly and Annotation of the Genome of a California Endemic Oak *Quercus lobata* Née (Fagaceae)." *G3 (Bethesda)* 6 (2016): 3485-95.

189. Plomion, C., J.-M. Aury, J. Amselem, T. Leroy, F. Murat, S. Duplessis, S. Faye, N. Francillonne, K. Labadie, G. Le Provost*, et al.* "Oak genome reveals facets of long lifespan." *Nat Plants* 4 (2018): 440-52.

190. Ramos, A. M., A. Usié, P. Barbosa, P. M. Barros, T. Capote, I. Chaves, F. Simões, I. Abreu, I. Carrasquinho, C. Faro*, et al.* "The draft genome sequence of cork oak." *Sci. Data* 5 (2018): 180069.

191. Fan, Y., S. K. Sahu, T. Yang, W. Mu, J. Wei, L. Cheng, J. Yang, R. Mu, J. Liu, J. Zhao*, et al.* "Dissecting the genome of star fruit (*Averrhoa carambola* L.)." *Hortic Res* 7 (2020): s41438-020-0306-4.

192. Wu, S., W. Sun, Z. Xu, J. Zhai, X. Li, C. Li, D. Zhang, X. Wu, L. Shen, J. Chen*, et al.* "The genome sequence of star fruit (*Averrhoa carambola*)." *Hortic Res* 7 (2020): s41438-020-0307-3.

193. Ming, R., S. Hou, Y. Feng, Q. Yu, A. Dionne-Laporte, J. H. Saw, P. Senin, W. Wang, B. V. Ly, K. L. T. Lewis*, et al.* "The draft genome of the transgenic tropical fruit tree papaya (*Carica papaya* Linnaeus)." *Nature* 452 (2008): 991-96.

194. Gao, Y., H. Wang, C. Liu, H. Chu, D. Dai, S. Song, L. Yu, L. Han, Y. Fu, B. Tian*, et al.* "De novo genome assembly of the red silk cotton tree (*Bombax ceiba*)." *GigaScience* 7 (2018): giy051.

195. Teh, B. T., K. Lim, C. H. Yong, C. C. Y. Ng, S. R. Rao, V. Rajasegaran, W. K. Lim, C. K. Ong, K. Chan, V. K. Y. Cheng*, et al.* "The draft genome of tropical fruit durian (*Durio zibethinus*)." *Nat Genet* 49 (2017): 1633-41.

196. Grover, C. E., D. Yuan, M. A. Arick, E. R. Miller, G. Hu, D. G. Peterson, J. F. Wendel and J. A. Udall. "The *Gossypium anomalum* genome as a resource for cotton improvement and evolutionary analysis of hybrid incompatibility." *G3 (Bethesda)* (2021): jkab319.

197. Li, F., G. Fan, K. Wang, F. Sun, Y. Yuan, G. Song, Q. Li, Z. Ma, C. Lu, C. Zou*, et al.* "Genome sequence of the cultivated cotton *Gossypium arboreum*." *Nat Genet* 46 (2014): 567-72.

198. Huang, G., Z. Wu, R. G. Percy, M. Bai, Y. Li, J. E. Frelichowski, J. Hu, K. Wang, J. Z. Yu and Y. Zhu. "Genome sequence of *Gossypium herbaceum* and genome updates of *Gossypium arboreum* and *Gossypium hirsutum* provide insights into cotton A-genome evolution." *Nat Genet* 52 (2020): 516-24.

199. Cai, Y., X. Cai, Q. Wang, P. Wang, Y. Zhang, C. Cai, Y. Xu, K. Wang, Z. Zhou, C. Wang*, et al.* "Genome sequencing of the Australian wild diploid species *Gossypium australe* highlights disease resistance and delayed gland morphogenesis." *Plant Biotechnol J* 18 (2020): 814-28.

200. Liu, X., B. Zhao, H.-J. Zheng, Y. Hu, G. Lu, C.-Q. Yang, J.-D. Chen, J.-J. Chen, D.-Y. Chen, L. Zhang*, et al.* "*Gossypium barbadense* genome sequence provides insight into the evolution of extra-long staple fiber and specialized metabolites." *Sci Rep-Uk* 5 (2015): 14139.

201. Hu, Y., J. Chen, L. Fang, Z. Zhang, W. Ma, Y. Niu, L. Ju, J. Deng, T. Zhao, J. Lian*, et al.* "*Gossypium barbadense* and *Gossypium hirsutum* genomes provide insights into the origin and evolution of allotetraploid cotton." *Nat Genet* 51 (2019): 739-48.
202. Chen, Z. J., A. Sreedasyam, A. Ando, Q. Song, L. M. De Santiago, A. M. Hulse-Kemp, M. Ding, W. Ye, R. C. Kirkbride, J. Jenkins*, et al.* "Genomic diversifications of five Gossypium allopolyploid species and their impact on cotton improvement." *Nat Genet* 52 (2020): 525-33.
203. Ma, Z., Y. Zhang, L. Wu, G. Zhang, Z. Sun, Z. Li, Y. Jiang, H. Ke, B. Chen, Z. Liu*, et al.* "High-quality genome assembly and resequencing of modern cotton cultivars provide resources for crop improvement." *Nat Genet* 53 (2021): 1385-91.
204. Li, F., G. Fan, C. Lu, G. Xiao, C. Zou, R. J. Kohel, Z. Ma, H. Shang, X. Ma, J. Wu*, et al.* "Genome sequence of cultivated Upland cotton (*Gossypium hirsutum* TM-1) provides insights into genome evolution." *Nat Biotechnol* 33 (2015): 524-30.
205. Grover, C. E., M. Pan, D. Yuan, M. A. Arick, G. Hu, L. Brase, D. M. Stelly, Z. Lu, R. J. Schmitz, D. G. Peterson*, et al.* "The *Gossypium longicalyx* Genome as a Resource for Cotton Breeding and Evolution." *G3 (Bethesda)* 10 (2020): 1457-67.
206. Wang, K., Z. Wang, F. Li, W. Ye, J. Wang, G. Song, Z. Yue, L. Cong, H. Shang, S. Zhu*, et al.* "The draft genome of a diploid cotton *Gossypium raimondii*." *Nat Genet* 44 (2012): 1098-103.
207. Udall, J. A., E. Long, C. Hanson, D. Yuan, T. Ramaraj, J. L. Conover, L. Gong, M. A. Arick, C. E. Grover, D. G. Peterson*, et al.* "De Novo Genome Sequence Assemblies of *Gossypium raimondii* and *Gossypium turneri*." *G3 (Bethesda)* 9 (2019): 3079-85.
208. Shen, C., N. Wang, Zhu, P. Wang, M. Wang, T. Wen, Y. Le, M. Wu, T. Yao, X. Zhang*, et al.* "*Gossypium tomentosum* genome and interspecific ultra-dense genetic maps reveal genomic structures, recombination landscape and flowering depression in cotton." *Genomics* 113 (2021): 1999-2009.
209. Argout, X., J. Salse, J.-M. Aury, M. J. Guiltinan, G. Droc, J. Gouzy, M. Allegre, C. Chaparro, T. Legavre, S. N. Maximova*, et al.* "The genome of *Theobroma cacao*." *Nat Genet* 43 (2011): 101-08.
210. Chen, C.-H., T. C.-Y. Kuo, M.-H. Yang, T.-Y. Chien, M.-J. Chu, L.-C. Huang, C.-Y. Chen, H.-F. Lo, S.-T. Jeng and L.-F. O. Chen. "Identification of cucurbitacins and assembly of a draft genome for *Aquilaria agallocha*." *BMC Genomics* 15 (2014): 578.
211. Ding, X., W. Mei, Q. Lin, H. Wang, J. Wang, S. Peng, H. Li, J. Zhu, W. Li, P. Wang*, et al.* "Genome sequence of the agarwood tree *Aquilaria sinensis* (Lour.) Spreng: the first chromosome-level draft genome in the Thymelaeceae family." *GigaScience* 9 (2020): giaa013.
212. Nong, W., S. T. S. Law, A. Y. P. Wong, T. Baril, T. Swale, L. M. Chu, A. Hayward, D. T. W. Lau and J. H. L. Hui. "Chromosomal-level reference genome of the incense tree *Aquilaria sinensis*." *Mol. Ecol. Resour.* 20 (2020): 971-79.
213. Sun, W. H., Z. Li, S. Xiang, L. Ni, D. Zhang, D. Q. Chen, M. Y. Qiu, Q. G. Zhang, L. Xiao, L. Din*, et al.* "The *Euscaphis japonica* genome and the evolution of malvids." *Plant J* (2021): tpj.15518.
214. Zhao, P., G. Xin, F. Yan, H. Wang, X. Ren, K. Woeste and W. Liu. "The de novo genome assembly of *Tapiscia sinensis* and the transcriptomic and developmental bases of androdioecy." *Hortic Res* 7 (2020): s41438-020-00414-w.
215. Healey, A. L., M. Shepherd, G. J. King, J. B. Butler, J. S. Freeman, D. J. Lee, B. M. Potts, O. B. Silva-Junior, A. Baten, J. Jenkins*, et al.* "Pests, diseases, and aridity have shaped the genome of *Corymbia citriodora*." *Commun Biol* 4 (2021): s42003-021-02009-0.
216. Hirakawa, H., Y. Nakamura, T. Kaneko, S. Isobe, H. Sakai, T. Kato, T. Hibino, S. Sasamoto, A. Watanabe, M. Yamada*, et al.* "Survey of the genetic information carried in the genome of *Eucalyptus camaldulensis*." *Plant Biotechnology* 28 (2011): 471-80.
217. Myburg, A. A., D. Grattapaglia, G. A. Tuskan, U. Hellsten, R. D. Hayes, J. Grimwood, J. Jenkins, E. Lindquist, H. Tice, D. Bauer*, et al.* "The genome of *Eucalyptus grandis*." *Nature* 510 (2014): 356-62.

218. Wang, W., A. Das, D. Kainer, M. Schalamun, A. Morales-Suarez, B. Schwessinger and R. Lanfear. "The draft nuclear genome assembly of *Eucalyptus pauciflora*: a pipeline for comparing de novo assemblies." *GigaScience* 9 (2020): giz160.
219. Voelker, J., M. Shepherd and R. Mauleon. "A high-quality draft genome for *Melaleuca alternifolia* (tea tree): a new platform for evolutionary genomics of myrtaceous terpene-rich species." *Gigabyte* 2021 (2021): 1-15.
220. Feng, C., C. Feng, X. Lin, S. Liu, Y. Li and M. Kang. "A chromosome-level genome assembly provides insights into ascorbic acid accumulation and fruit softening in guava (*Psidium guajava*)." *Plant Biotechnol J* 19 (2021): 717-30.
221. Yuan, Z., Y. Fang, T. Zhang, Z. Fei, F. Han, C. Liu, M. Liu, W. Xiao, W. Zhang, S. Wu*, et al.* "The pomegranate (*Punica granatum* L.) genome provides insights into fruit quality and ovule developmental biology." *Plant Biotechnol J* 16 (2018): 1363-74.
222. Luo, X., H. Li, Z. Wu, W. Yao, P. Zhao, D. Cao, H. Yu, K. Li, K. Poudel, D. Zhao*, et al.* "The pomegranate (*Punica granatum* L.) draft genome dissects genetic divergence between soft- and hard-seeded cultivars." *Plant Biotechnol J* 18 (2020): 955-68.
223. Qin, G., C. Xu, R. Ming, H. Tang, R. Guyot, E. M. Kramer, Y. Hu, X. Yi, Y. Qi, X. Xu*, et al.* "The pomegranate (*Punica granatum* L.) genome and the genomics of punicalagin biosynthesis." *Plant J* 91 (2017): 1108-28.
224. Wang, X., Y. Xu, S. Zhang, L. Cao, Y. Huang, J. Cheng, G. Wu, S. Tian, C. Chen, Y. Liu*, et al.* "Genomic analyses of primitive, wild and cultivated citrus provide insights into asexual reproduction." *Nat Genet* 49 (2017): 765-72.
225. Wu, G. A., S. Prochnik, J. Jenkins, J. Salse, U. Hellsten, F. Murat, X. Perrier, M. Ruiz, S. Scalabrin, J. Terol*, et al.* "Sequencing of diverse mandarin, pummelo and orange genomes reveals complex history of admixture during citrus domestication." *Nat Biotechnol* 32 (2014): 656-62.
226. Zhang, Y., G. Barthe, J. W. Grosser and N. Wang. "Transcriptome analysis of root response to citrus blight based on the newly assembled *Swingle citrumelo* draft genome." *BMC Genomics* 17 (2016): s12864-016-2779-y.
227. Wang, L., F. He, Y. Huang, J. He, S. Yang, J. Zeng, C. Deng, X. Jiang, Y. Fang, S. Wen*, et al.* "Genome of Wild Mandarin and Domestication History of Mandarin." *Mol Plant* 11 (2018): 1024-37.
228. Xu, Q., L.-L. Chen, X. Ruan, D. Chen, A. Zhu, C. Chen, D. Bertrand, W.-B. Jiao, B.-H. Hao, M. P. Lyon*, et al.* "The draft genome of sweet orange (*Citrus sinensis*)." *Nat Genet* 45 (2013): 59-66.
229. Shimizu, T., Y. Tanizawa, T. Mochizuki, H. Nagasaki, T. Yoshioka, A. Toyoda, A. Fujiyama, E. Kaminuma and Y. Nakamura. "Draft Sequencing of the Heterozygous Diploid Genome of Satsuma (*Citrus unshiu* Marc.) Using a Hybrid Assembly Approach." *Front Genet* 8 (2017): 180.
230. Peng, Z., J. V. Bredeson, G. A. Wu, S. Shu, N. Rawat, D. Du, S. Parajuli, Q. Yu, Q. You, D. S. Rokhsar*, et al.* "A chromosome-scale reference genome of trifoliate orange (*Poncirus trifoliata*) provides insights into disease resistance, cold tolerance and genome evolution in Citrus." *Plant J* 104 (2020): 1215-32.
231. Feng, S., Z. Liu, J. Cheng, Z. Li, L. Tian, M. Liu, T. Yang, Y. Liu, Y. Liu, H. Dai*, et al.* "Zanthoxylum-specific whole genome duplication and recent activity of transposable elements in the highly repetitive paleotetraploid *Z. bungeanum* genome." *Hortic Res* 8 (2021): s41438-021-00665-1.
232. Krishnan, N. M., S. Pattnaik, P. Jain, P. Gaur, R. Choudhary, S. Vaidyanathan, S. Deepak, A. K. Hariharan, P. Krishna, J. Nair*, et al.* "A draft of the genome and four transcriptomes of a medicinal and pesticidal angiosperm *Azadirachta indica*." *BMC Genomics* 13 (2012): 464.
233. Ji, Y. T., Z. Xiu, C. H. Chen, Y. Wang, J. X. Yang, J. J. Sui, S. J. Jiang, P. Wang, S. Y. Yue, Q. Q. Zhang*, et al.* "Long read sequencing of *Toona sinensis* (A. Juss) Roem: A chromosome-level reference genome for the family Meliaceae." *Mol Ecol Resour* 21 (2021): 1243-55.

234. Wang, P., Y. Luo, J. Huang, S. Gao, G. Zhu, Z. Dang, J. Gai, M. Yang, M. Zhu, H. Zhang*, et al.* "The genome evolution and domestication of tropical fruit mango." *Genome Biol* 21 (2020): s13059-020-01959-8.
235. Bally, I. S. E., A. Bombarely, A. H. Chambers, Y. Cohen, N. L. Dillon, D. J. Innes, M. A. Islas-Osuna, D. N. Kuhn, L. A. Mueller, R. Ophir*, et al.* "The 'Tommy Atkins' mango genome reveals candidate genes for fruit quality." *BMC Plant Biology* 21 (2021):
236. Zeng, L., X.-L. Tu, H. Dai, F.-M. Han, B.-S. Lu, M.-S. Wang, H. A. Nanaei, A. Tajabadipour, M. Mansouri, X.-L. Li*, et al.* "Whole genomes and transcriptomes reveal adaptation and domestication of pistachio." *Genome Biol* 20 (2019): s13059-019-1686-3.
237. Ma, Q., T. Sun, S. Li, J. Wen, L. Zhu, T. Yin, K. Yan, X. Xu, S. Li, J. Mao*, et al.* "The *Acer truncatum* genome provides insights into nervonic acid biosynthesis." *Plant J* 104 (2020): 662-78.
238. Yang, J., H. M. Wariss, L. Tao, R. Zhang, Q. Yun, P. Hollingsworth, Z. Dao, G. Luo, H. Guo, Y. Ma*, et al.* "De novo genome assembly of the endangered *Acer yangbiense*, a plant species with extremely small populations endemic to Yunnan Province, China." *GigaScience* 8 (2019): giz085.
239. Lin, Y., J. Min, R. Lai, Z. Wu, Y. Chen, L. Yu, C. Cheng, Y. Jin, Q. Tian, Q. Liu*, et al.* "Genome-wide sequencing of longan (*Dimocarpus longan* Lour.) provides insights into molecular basis of its polyphenol-rich characteristics." *GigaScience* 6 (2017): gix023.
240. Zhang, W., J. Lin, J. Li, S. Zheng, X. Zhang, S. Chen, X. Ma, F. Dong, H. Jia, X. Xu*, et al.* "Rambutan genome revealed gene networks for spine formation and aril development." *Plant J* (2021):
241. Liang, Q., H. Li, S. Li, F. Yuan, J. Sun, Q. Duan, Q. Li, R. Zhang, Y. L. Sang, N. Wang*, et al.* "The genome assembly and annotation of yellowhorn (*Xanthoceras sorbifolium* Bunge)." *GigaScience* 8 (2019): giz071.
242. Bi, Q., Y. Zhao, W. Du, Y. Lu, L. Gui, Z. Zheng, H. Yu, Y. Cui, Z. Liu, T. Cui*, et al.* "Pseudomolecule-level assembly of the Chinese oil tree yellowhorn (*Xanthoceras sorbifolium*) genome." *GigaScience* 8 (2019): giz070.
