# Supplementary material for: From Genome Sequencing to CRISPR-Based Genome Editing for Climate-Resilient Forest Trees
Source: Int J Mol Sci. 2022 Jan 16;23(2):966. doi: 10.3390/ijms23020966 (PMC8780650; doi:10.3390/ijms23020966)
Supplement: Supplementary file 1 [file ijms-23-00966-s001.zip › Table S2.pdf]

Supplemental Table S2: Detailed data of Figure 2: Overview of the constructed CRISPR/Cas systems used in genome editing of forest trees

**Species**	**Clone names**	**Gene targets**	**BI tools**	**Promoter-Cas9**	**Promoter-sgRNA**	**CRISPR system**	**Mutation rate (%)**	**Delivery**	**Purpose**	**Reference**
**Poplars / Aspen**										
Populus tomentosa	741	PDS	ZiFiT	pCaMV35S	pAtU3/U6	pSpCas9	52%	sAg	Concept	[1]
Populus tremula x alba	717-1B4	4CL1,2,5	CRISPR-P	pCaMV35S (2x)	pMtU6.6	hSpCas9	n.d.	sAg	Growth	[2]
Populus tomentosa	741	WRKY18, 35	ZiFiT	pCaMV35S	pAtU3/U6	pSpCas9	n.d.	sAg	Disease	[3]
Populus tomentosa	741	MYB57	ZiFiT	pCaMV35S	pAtU3/U6	pSpCas9	n.d.	sAg	Disease	[4]
Populus tomentosa	741	MYB115	ZiFiT	pCaMV35S	pAtU3/U6	pSpCas9	93 – 100%	sAg	Disease	[5]
Populus tomentosa	741	MYB170	ZiFiT	pCaMV35S	pAtU3/U6	pSpCas9	64%	sAg	Lignin	[6]
Populus tomentosa	741	MYB156	ZiFiT	pCaMV35S	pAtU3/U6	pSpCas9	48%	sAg	Lignin	[7]
Populus tremula × alba	717-1B4	LFY, AG1, AG2	ZiFiT	pCaMV35S (2x)	pAtU6-26	hSpCas9	0 – 79%	sAg	Flower	[8]
P. tremula × tremuloides	353-38	LFY, AG1, AG2	ZiFiT	pCaMV35S (2x)	pAtU6-26	hSpCas9	46 – 81%	sAg	Flower	[8]
Populus tomentosa	741	JMJ25	ZiFiT	pCaMV35S	pAtU3/U6	pSpCas9	82%	sAg	Disease	[9]
Populus tremula × alba	717-1B4	BRC1, 2	AspenDB	pCaMV35S	pAtU3	pSpCas9	70 – 90%	sAg	Growth	[10]
Populus tremula × alba	717-1B4	SOC1, AG		pCaMV35S	pAtU6	SpCas9	1 – 98%	sAg	Flower	[11]
Populus tremula × alba	717-1B4	NFP		pCaMV35S	pAtU6	SpCas9	1 – 100%	sAg	Function	[11]
Populus tremula	W52	TOZ19		pCaMV35S	pAtU6	SpCas9	62%	sAg	Sex	[11]
Populus alba	pyramidalis	C2H2-AZF		pCaMV35S	pAtU3/U6	pSpCas9	89%	sAg	Concept	[12]
Populus tremula L. × tremuloides	T89	VNS		pPcUbi4-2	pAtU6-26	aSpCas9	80%	sAg	Wood	[13]
Populus alba x glandulosa	84 K	PDS		pCaMV35S (2x)	pAtU6-26	AsCas12a	57-70%	sAg	Concept	[14]

Populus alba x glandulosa	84 K	PDS		pCaMV35S (2x)	pAtU6-26	LbCas12a	30-33%	sAg	Concept	[14]
Populus alba x glandulosa	84 K	PDS		pCaMV35S (2x)	pAtU6-26	FnCas12a	3-7%	sAg	Concept	[14]
Populus tremula × alba	717-1B4	CCR2	AspenDB	pCaMV35S (2x)	pMtU6.6	hSpCas9	100%	sAg	Lignin	[15]
Populus tremula L. × tremuloides	T89	BRC1	CRISPR-P	pCaMV35S	pAtU6-26	zSpCas9	n.d.	sAg	Growth	[16]
Populus tremula	W52	ARR17	CRISPR-P	pCaMV35S	pAtU6	SpCas9	n.d.	sAg	Sex	[17]
Populus davidiana × bolleana	Shanxin yang	PDS	CRISPR-GE	pCaMV35S (2x)	pAtU6-26, 29	zSpCas9	86%	sAg	Concept	[18]
Populus alba x glandulosa	84 K	NF-YB21	CRISPR-P	pPcUbi4-2	pAtU6	aSpCas9	85%	sAg	Stress	[19]
Populus tremula × alba	717-1B4	SAP	AspenDB	pZmUbi	pAtU3d	pSpCas9	55%	sAg	Flower	[20]
P. tremula × tremuloides	353-38	SAP	AspenDB	pZmUbi	pAtU3d	pSpCas9	55%	sAg	Flower	[20]
Populus alba x glandulosa		PDS	CRISPR RGEN	pCaMV35S	pAtU6-26	zSpCas9	75%	sAg	Concept	[21]
Populus tremula x alba	717-1B4	CCR2	AspenDB	pCaMV35S (2x)	pMtU6.6	hSpCas9-transgene	n.d.	sAg	Lignin	[22]
Populus tremula x alba	717-1B4	CSE	AspenDB	pCaMV35S (2x)	pMtU6.6	hSpCas9	95%	sAg	Lignin	[23]
Populus tremula L. × tremuloides	T89	PHYB1,2		pCaMV35S	pAtU6	aSpCas9	n.d.	sAg	Growth	[24]
Populus tomentosa	741	BBX23	CRISPR-GE	pCaMV35S	pAtU3/U6	pSpCas9	96-100%	sAg	Disease	[25]
**Eucalypts**										
Eucalyptus grandis	LCFA001	CCR1	CRISPOR	pCaMV35S (2x)	pAtU6	hSpCas9	100%	sAg	Lignin	[26]
Eucalyptus grandis	LCFA001	IAA9A	CRISPOR	pCaMV35S	pAtU6	hSpCas9	92%	sAg	Wood	[26]

				(2x)						
Eucalyptus grandis x urophylla	SP7	LFY	ZiFiT	pCaMV35S (2x)	pAtU6-26	hSpCas9	100%	sAg	Flower	[27]
**Rubber tree**										
Hevea brasiliesis		FT, TFL1		direct	direct	RNP-Cas9	4 – 20%	PEG	Flower	[28]
Hevea brasiliesis		FT, TFL1		pCaMV35S (2x)	pHbU6	oSpCas9	19%	sAg	Flower	[29]
**Conifers**										
Pinus radiata		GUX1		direct	direct	RNP-Cas9	15-33%	PB	Concept	[30]

Target genes: 4CL: 4-Coumarate:CoA ligase; PDS: phytoene desaturase; LFY: Leafy; AG(L): Agamous(-like) genes; SOC1: Suppressor of Overexpression of Constans1; FUL: Fruitful; NFP: Nod-Factor Perception; TOZ19: Tormozembryo Defective 19; BRC = Branched; VNS: Wood-associated NAC domain TFs; CCR1: Cinnamoyl CoA reductase1; IAA9A: Aux/IAA transcription factor; SAP: Sterile Apetala ; FT: Flowering Locus T; TFL1: Terminal Flower1; PHYB1,2: Phytocrome B; GUX1: Glucuronic acid Substitution of the Xylan 1

BI tools: Bioinformatics tools for sgRNA design and off-target detection. ZiFiT: ZiFiT Targeter v4.2 (zifit.partners.org/ZiFiT [31]); CRISPR-P: crispr.hzau.edu.cn/CRISPR2/ [32]; CRISPOR: crispor.tefor.net [33]; CRISPR RGEN: rgenome.net [34]; CRISPR-GE: skl.scau.edu.cn [35]; n.d. : not determined

Promoters: pCaMV35S: The very strong constitutive promoter of the cauliflower mosaic virus (CaMV) 35S RNA; pUbi4-2: constitutive Ubiquitin4–2 promoter from *Petroselinum crispum*; pAtU3/U6: Arabidopsis pAtU3b,d or pAtU6-1,29 promoters; pMtU6.6 : Medicago promoter; pHbU6: endogenous H. brasiliesis U6 promoter.

CRISPR systems: SpCas9: *Streptococcus pyogenes* Cas9 [36]; aSpCas9, pSpCas9, zSpCas9, oSpCas9, and hSpCas9, : *S. pyogenes* Cas9 with codon optimization for *Arabidopsis thaliana* [37], for plant [38], for maize [39], for rice [40] and for human [41, 42], respectively. AsCas12a, LbCas12a, and FnCas12a: Cas12 from

Delivery: sAg: stable Agrobacterium-mediated transformation; PEG: Polyethylene glycol (PEG)-mediated transformation; PB: particle bombardment transformaion

Purpose: Concept: Proof-of-Concept studies; Disease: Disease resistance; Lignin: Lignin modification; Flower: Flowering control; Growth: Growth enhancement; Sex: Sex determination; Function: Gene function studies; Wood: Wood composition; Stress: Abiotic or biotic stress tolerance

References:

1. Fan, D., T. Liu, C. Li, B. Jiao, S. Li, Y. Hou and K. Luo. "Efficient CRISPR/Cas9-mediated targeted mutagenesis in Populus in the first generation." *Sci Rep* 5 (2015): 12217.
2. Zhou, X., T. B. Jacobs, L. J. Xue, S. A. Harding and C. J. Tsai. "Exploiting SNPs for biallelic CRISPR mutations in the outcrossing woody perennial Populus reveals 4-coumarate:CoA ligase specificity and redundancy." *New Phytol* 208 (2015): 298-301.
3. Jiang, Y., L. Guo, X. Ma, X. Zhao, B. Jiao, C. Li and K. Luo. "The WRKY transcription factors PtrWRKY18 and PtrWRKY35 promote Melampsora resistance in Populus." *Tree Physiol.* 37 (2017): 665-75.
4. Wan, S., C. Li, X. Ma and K. Luo. "PtrMYB57 contributes to the negative regulation of anthocyanin and proanthocyanidin biosynthesis in poplar." *Plant Cell Rep* 36 (2017): 1263-76.
5. Wang, L., L. Ran, Y. Hou, Q. Tian, C. Li, R. Liu, D. Fan and K. Luo. "The transcription factor MYB115 contributes to the regulation of proanthocyanidin biosynthesis and enhances fungal resistance in poplar." *New Phytol* 215 (2017): 351-67.
6. Xu, C., X. Fu, R. Liu, L. Guo, L. Ran, C. Li, Q. Tian, B. Jiao, B. Wang and K. Luo. "PtoMYB170 positively regulates lignin deposition during wood formation in poplar and confers drought tolerance in transgenic Arabidopsis." *Tree Physiol.* 37 (2017): 1713-26.
7. Yang, L., X. Zhao, L. Ran, C. Li, D. Fan and K. Luo. "PtoMYB156 is involved in negative regulation of phenylpropanoid metabolism and secondary cell wall biosynthesis during wood formation in poplar." *Sci Rep-Uk* 7 (2017): 41209.
8. Elorriaga, E., A. L. Klocko, C. Ma and S. H. Strauss. "Variation in Mutation Spectra Among CRISPR/Cas9 Mutagenized Poplars." *Front Plant Sci* 9 (2018): 594.
9. Fan, D., X. Wang, X. Tang, X. Ye, S. Ren, D. Wang and K. Luo. "Histone H3K9 demethylase JMJ25 epigenetically modulates anthocyanin biosynthesis in poplar." *Plant J* 96 (2018): 1121-36.
10. Muhr, M., M. Paulat, M. Awwanah, M. Brinkkötter and T. Teichmann. "CRISPR/Cas9-mediated knockout of Populus BRANCHED1 and BRANCHED2 orthologs reveals a major function in bud outgrowth control." *Tree Physiol.* 38 (2018): 1588-97.
11. Bruegmann, T., K. Deecke and M. Fladung. "Evaluating the Efficiency of gRNAs in CRISPR/Cas9 Mediated Genome Editing in Poplars." *Int J Mol Sci* 20 (2019): 3623.
12. Ma, J., D. Wan, B. Duan, X. Bai, Q. Bai, N. Chen and T. Ma. "Genome sequence and genetic transformation of a widely distributed and cultivated poplar." *Plant Biotechnol J* 17 (2019): 451-60.
13. Takata, N., T. Awano, M. T. Nakata, Y. Sano, S. Sakamoto, N. Mitsuda and T. Taniguchi. "Populus NST/SND orthologs are key regulators of secondary cell wall formation in wood fibers, phloem fibers and xylem ray parenchyma cells." *Tree Physiol.* 39 (2019): 514-25.
14. An, Y., Y. Geng, J. Yao, C. Fu, M. Lu, C. Wang and J. Du. "Efficient Genome Editing in Populus Using CRISPR/Cas12a." *Front Plant Sci* 11 (2020): 593938.
15. De Meester, B., B. Madariaga Calderón, L. De Vries, J. Pollier, G. Goeminne, J. Van Doorsselaere, M. Chen, J. Ralph, R. Vanholme and W. Boerjan. "Tailoring poplar lignin without yield penalty by combining a null and haploinsufficient CINNAMOYL-CoA REDUCTASE2 allele." *Nat Commun* 11 (2020): s41467-020-18822-w.
16. Maurya, J. P., R. K. Singh, P. C. Miskolczi, A. N. Prasad, K. Jonsson, F. Wu and R. P. Bhalerao. "Branching Regulator BRC1 Mediates Photoperiodic Control of Seasonal Growth in Hybrid Aspen." *Current Biology* 30 (2020): 122-26.e2.
17. Müller, N. A., B. Kersten, A. P. Leite Montalvão, N. Mähler, C. Bernhardsson, K. Bräutigam, Z. Carracedo Lorenzo, H. Hoenicka, V. Kumar, M. Mader*, et al.* "A single gene underlies the dynamic evolution of poplar sex determination." *Nat Plants* 6 (2020): 630-37.
18. Wang, J., H. T. Wu, Y. N. Chen and T. M. Yin. "Efficient CRISPR/Cas9-Mediated Gene Editing in an Interspecific Hybrid Poplar With a Highly Heterozygous Genome." *Front Plant Sci* 11 (2020): fpls.2020.00996.
19. Zhou, Y., Y. Zhang, X. Wang, X. Han, Y. An, S. Lin, C. Shen, J. Wen, C. Liu, W. Yin*, et al.* "Root-specific NF-Y family transcription factor, PdNF-YB21 , positively regulates root

growth and drought resistance by abscisic acid-mediated indoylacetic acid transport in Populus." *New Phytol* 227 (2020): 407-26.
20. Azeez, A. and V. Busov. "CRISPR/Cas9-mediated single and biallelic knockout of poplar STERILE APETALA (PopSAP) leads to complete reproductive sterility." *Plant Biotechnol J* 19 (2021): 23-25.
21. Bae, E.-K., H. Choi, J. W. Choi, H. Lee, S.-G. Kim, J.-H. Ko and Y.-I. Choi. "Efficient knockout of the phytoene desaturase gene in a hybrid poplar (*Populus alba* × *Populus glandulosa*) using the CRISPR/Cas9 system with a single gRNA." *Transgenic Res* (2021): s11248-021-00272-9.
22. De Meester, B., R. Vanholme, L. De Vries, M. Wouters, J. Van Doorsselaere and W. Boerjan. "Vessel- and ray-specific monolignol biosynthesis as an approach to engineer fiber-hypolignification and enhanced saccharification in poplar." *Plant J* (2021): tpj.15468.
23. De Vries, L., M. Brouckaert, A. Chanoca, H. Kim, M. R. Regner, V. I. Timokhin, Y. Sun, B. De Meester, J. Van Doorsselaere, G. Goeminne*, et al.* "CRISPR-Cas9 editing of CAFFEOYL SHIKIMATE ESTERASE 1 and 2 shows their importance and partial redundancy in lignification in *Populus tremula* × *P. alba*." *Plant Biotechnol J* (2021): pbi.13651.
24. Ding, J., B. Zhang, Y. Li, D. André and O. Nilsson. "Phytochrome B and PHYTOCHROME INTERACTING FACTOR8 modulate seasonal growth in trees." *New Phytol* (2021): nph.17350.
25. Li, C., J. Pei, X. Yan, X. Cui, M. Tsuruta, Y. Liu and C. Lian. "A poplar B-box protein PtrBBX23 modulates the accumulation of anthocyanins and proanthocyanidins in response to high light." *Plant, Cell & Environment* 44 (2021): 3015-33.
26. Dai, Y., G. Hu, A. Dupas, L. Medina, N. Blandels, H. San Clemente, N. Ladouce, M. Badawi, G. Hernandez-Raquet, F. Mounet*, et al.* "Implementing the CRISPR/Cas9 Technology in Eucalyptus Hairy Roots Using Wood-Related Genes." *Int J Mol Sci* 21 (2020): 3408.
27. Elorriaga, E., A. L. Klocko, C. Ma, M. Plessis, X. An, A. A. Myburg and S. H. Strauss. "Genetic containment in vegetatively propagated forest trees: CRISPR disruption of LEAFY function in Eucalyptus gives sterile indeterminate inflorescences and normal juvenile development." *Plant Biotechnol J* 19 (2021): 1743-55.
28. Fan, Y., S. Xin, X. Dai, X. Yang, H. Huang and Y. Hua. "Efficient genome editing of rubber tree (*Hevea brasiliensis*) protoplasts using CRISPR/Cas9 ribonucleoproteins." *Ind Crop Prod* 146 (2020): 112146.
29. Dai, X., X. Yang, C. Wang, Y. Fan, S. Xin, Y. Hua, K. Wang and H. Huang. "CRISPR/Cas9-mediated genome editing in *Hevea brasiliensis*." *Ind Crop Prod* 164 (2021): 113418.
30. Poovaiah, C., L. Phillips, B. Geddes, C. Reeves, M. Sorieul and G. Thorlby. "Genome editing with CRISPR/Cas9 in *Pinus radiata* (D. Don)." *BMC Plant Biology* 21 (2021): 363.
31. Sander, J. D., M. L. Maeder, D. Reyon, D. F. Voytas, J. K. Joung and D. Dobbs. "ZiFiT (Zinc Finger Targeter): an updated zinc finger engineering tool." *Nucleic Acids Res* 38 (2010): W462-8.
32. Liu, H., Y. Ding, Y. Zhou, W. Jin, K. Xie and L. L. Chen. "CRISPR-P 2.0: An Improved CRISPR-Cas9 Tool for Genome Editing in Plants." *Mol Plant* 10 (2017): 530-32.
33. Haeussler, M., K. Schonig, H. Eckert, A. Eschstruth, J. Mianne, J. B. Renaud, S. Schneider-Maunoury, A. Shkumatava, L. Teboul, J. Kent*, et al.* "Evaluation of off-target and on-target scoring algorithms and integration into the guide RNA selection tool CRISPOR." *Genome Biol* 17 (2016): 148.
34. Park, J., S. Bae and J. S. Kim. "Cas-Designer: a web-based tool for choice of CRISPR-Cas9 target sites." *Bioinformatics* 31 (2015): 4014-6.
35. Xie, X., X. Ma, Q. Zhu, D. Zeng, G. Li and Y.-G. Liu. "CRISPR-GE: A Convenient Software Toolkit for CRISPR-Based Genome Editing." *Mol Plant* 10 (2017): 1246-49.
36. Jinek, M., K. Chylinski, I. Fonfara, M. Hauer, J. A. Doudna and E. Charpentier. "A programmable dual-RNA-guided DNA endonuclease in adaptive bacterial immunity." *Science* 337 (2012): 816-21.
37. Fauser, F., S. Schiml and H. Puchta. "Both CRISPR/Cas-based nucleases and nickases can be used efficiently for genome engineering in *Arabidopsis thaliana*." *Plant J* 79 (2014): 348-59.

38. Ma, X., Q. Zhang, Q. Zhu, W. Liu, Y. Chen, R. Qiu, B. Wang, Z. Yang, H. Li, Y. Lin*, et al.* "A Robust CRISPR/Cas9 System for Convenient, High-Efficiency Multiplex Genome Editing in Monocot and Dicot Plants." *Mol Plant* 8 (2015): 1274-84.
39. Xing, H.-L., L. Dong, Z.-P. Wang, H.-Y. Zhang, C.-Y. Han, B. Liu, X.-C. Wang and Q.-J. Chen. "A CRISPR/Cas9 toolkit for multiplex genome editing in plants." *BMC Plant Biology* 14 (2014): s12870-014-0327-y.
40. Shan, Q. W., Y. P. Wang, J. Li, Y. Zhang, K. L. Chen, Z. Liang, K. Zhang, J. X. Liu, J. J. Xi, J. L. Qiu*, et al.* "Targeted genome modification of crop plants using a CRISPR-Cas system." *Nat Biotechnol* 31 (2013): 686-88.
41. Mali, P., L. Yang, K. M. Esvelt, J. Aach, M. Guell, J. E. DiCarlo, J. E. Norville and G. M. Church. "RNA-guided human genome engineering via Cas9." *Science* 339 (2013): 823-6.
42. Cong, L., F. A. Ran, D. Cox, S. L. Lin, R. Barretto, N. Habib, P. D. Hsu, X. B. Wu, W. Y. Jiang, L. A. Marraffini*, et al.* "Multiplex genome engineering using CRISPR/Cas systems." *Science* 339 (2013): 819-23.
